# Heteroborospherene clusters Ni_n_ ∈ B_40_ (n = 1–4) and heteroborophene monolayers Ni_2_ ∈ B_14_ with planar heptacoordinate transition-metal centers in η^7^-B_7_ heptagons

**DOI:** 10.1038/s41598-017-06039-9

**Published:** 2017-07-18

**Authors:** Hai-Ru Li, Xin-Xin Tian, Xue-Mei Luo, Miao Yan, Yue-Wen Mu, Hai-Gang Lu, Si-Dian Li

**Affiliations:** Institute of Molecular Science, Shanxi University, Taiyuan, 030006 China

## Abstract

With inspirations from recent discoveries of the cage-like borospherene B_40_ and perfectly planar Co ∈ B_18_^−^ and based on extensive global minimum searches and first-principles theory calculations, we present herein the possibility of the novel planar Ni ∈ B_18_ (1), cage-like heteroborospherenes Ni_n_ ∈ B_40_ (n = 1–4) (2–5), and planar heteroborophenes Ni_2_ ∈ B_14_ (6, 7) which all contain planar or quasi-planar heptacoordinate transition-metal (phTM) centers in η^7^-B_7_ heptagons. The nearly degenerate Ni_2_ ∈ B_14_ (6) and Ni_2_ ∈ B_14_ (7) monolayers are predicted to be metallic in nature, with Ni_2_ ∈ B_14_ (6) composed of interwoven boron double chains with two phNi centers per unit cell being the precursor of cage-like Ni_n_ ∈ B_40_ (n = 1–4) (2–5). Detailed bonding analyses indicate that Ni_n_ ∈ B_40_ (n = 1–4) (2–5) and Ni_2_ ∈ B_14_ (6, 7) possess the universal bonding pattern of σ + π double delocalization on the boron frameworks, with each phNi forming three lone pairs in radial direction (3d_z2_^2^, 3d_zx_^2^, and 3d_yz_^2^) and two effective nearly in-plane 8c-2e σ-coordination bonds between the remaining tangential Ni 3d orbitals (3d_x2−y2_ and 3d_xy_) and the η^7^-B_7_ heptagon around it. The IR, Raman, and UV-vis absorption spectra of 1–5 are computationally simulated to facilitate their experimental characterizations.

## Introduction

Buckminsterfullerene C_60_ and its precursor graphene have been superstars in chemistry and materials science ever since their discoveries^1, 2^. As the light neighbor of carbon, boron has a rich chemistry dominated with multicenter two-electron bonds (*mc-2e* bonds) in both bulk allotropes and polyhedral molecules to compensate for its electron-deficiency^3^. Multicenter bonds also appear to be responsible for the planar or quasi-planar (2D) structures of an unprecedentedly wide range of boron clusters B_*n*_^−/0^ (n = 3−30, 35, 36) characterized in gas phase in the past decade, unveiling a flat world of boron analogous to polycyclic aromatic hydrocarbons^4–18^. The possibility of all-boron fullerenes was not considered until 2007 when the high-symmetry *I*_*h*_ B_80_ cage was constructed from C_60_ by capping the twenty hexagons^19^. However, this celebrated structure was later found to be much less stable than its core-shell rivals at various density functional theory (DFT) levels^20, 21^. The first all-boron fullerenes *D*_2*d*_ B_40_^−/0^, referred to as borospherenes in literature, were discovered in 2014 in a combined experimental and theoretical investigation^22^. In stark contrast to C_60_ which consists of twenty hexagons and twelve pentagons, *D*_2*d*_ B_40_ features four equivalent B_7_ heptagons with large cavities on the waist which may serve as effective ligands to coordinate transition metal centers. The first axially chiral borospherenes *C*_3_/*C*_2_ B_39_^−^ with three/four B_7_ heptagons were observed in 2015^23^. In the past two years, the B_n_^q^ (q = n−40) borospherene family has been expanded at first-principles theory level to include the cage-like B_41_^+^, B_42_^2+^ ^24^, B_40_^+^^25^, B_38_^2−^ (in Ca@B_38_)^26^, B_37_^3−^ (in Ca@B_37_^−^)^27^, and B_36_^4−^ (in Li_4_&B_36_)^28^ which are all composed of twelve interwoven boron double chains (BDCs) with six hexagonal and heptagonal faces on thesurface. These borospherenes follow the universal bonding pattern of σ + π double delocalization. Endohedral and exohedral charge-transfer complexes M@B_40_ (M = Ca, Sr, La, Y, Sc) and M&B_40_ (M = Be, Mg) were also predicted to be stable species^29, 30^. The experimentally observed quasi-planar B_36_^−/0^ and B_35_^−^ clusters with one or two adjacent hexagonal vacancies appeared to be molecular motifs of infinite 2D borophene monolayers^9, 10^. The first 2D borophene monolayers deposited on Ag(111) substrates were successfully synthesized by two independent groups in 2015^31, 32^. Interestingly, the most stable χ^3^-borophene deposited on Ag(111) is composed of zigzag BDCs in alternation with columns of adjacent hexagonal vacancies^32^, further indicating the importance of BDCs in low-dimensional boronanostructures. A boron monolayer with octagons, heptagons, and hexagons was recently considered to be a 2D structural precursor of the B_40_ cage^33^ though it is not a stable low-energy 2D polymorph compared to the well-known boron α-sheet and other low-lying structures^33–36^. Buckled FeB_2_ and FeB_6_ monolayers were recently proposed to be stable species with hexa-, hepta-, and octa-coordinate Fe centers^37, 38^. Furthermore, the recent observation of the perfectly planar *C*_*2v*_ Co ∈ B_18_^−^ with a planar heptacoordinate Co center opened vast opportunities to design 2D heteroborophene monolayers^39^ and other heteroboronanostructures with planar or quasi-planar heptacoordinate transition-metal (phTM) centers. However, to the best of our knowledge, cage-like heteroborospherenes with phTM centers on the surface and stable heteroborophene monolayers as precursors of such heteroborospherenes have not been reported in either experiments or theory.

Based on extensive global minimum (GM) searches and first-principles theory calculations, we predict herein the existence of the perfectly planar heteroborophene-type cluster Ni ∈ B_18_ (**1**), cage-like heteroborospherenes Ni_n_ ∈ B_40_ (n = 1–4) (**2**–**5**), and the nearly degenerate heteroborophene monolayers Ni_2_ ∈ B_14_ (**6**, **7**) which all contain phNi centers in η^7^-B_7_ heptagons on the boron frameworks, with Ni_2_ ∈ B_14_ (**6**) composed of interwoven BDCs being the structural precursor of the cage-like Ni_n_ ∈ B_40_ (n = 1–4) (**2**–**5**). These heteroboronanostructures possess the universal bonding pattern of σ + π double-delocalization on the boron frameworks, with each phNi center forming two effective 8c-2e σ-coordination bonds with the η^7^-B_7_ heptagon around it, inheriting the structural and bonding characteristics of the experimentally observed cage-like B_40_^22^ and perfectly planar Co ∈ B_18_^−^^39^.

### Computational procedures

Extensive GM searches were performed using both the Minima Hopping (MH)^40, 41^ and TGmin^42^ algorithms on NiB_18_ and Ni_n_B_40_ clusters (n = 1–2), in combination with manual structural constructions based on the low-lying isomers of CoB_18_^−^^39^ and B_40_^−/0^ ^22^. Low-lying structures were then fully optimized with frequencies checked at both PBE0^43^ and TPSSh^44^ levels with the 6–311 + G* basis set^45^ to ensure they are all true minima of the systems. The relative energies were further refined at the more accurate CCSD(T)^46–48^ level with 6–31 G* basis set for the five lowest-lying isomers of NiB_18_ and NiB_40_. A general global search based on the PSO technique implemented in the Crystal Structure Analysis by Particle Swarm Optimization (CALYPSO)^49^ package was performed on 2D Ni_2_B_14_ monolayer. The underlying plane-wave based DFT structrual optimization were performed using the Vienna *ab initio* simulation package (VASP)^50, 51^, within the framework of projector augmented wave (PAW) pseudopotential method^52, 53^ and PBE generalized gradient approximation (GGA)^54^. The Heyd−Scuseria−Ernzerhof (HSE06) approach^55^ was used to calculate the band structures and densities of states of the PBE structures.

## Results and Discussions

### Structures and Stabilities of Heteroborospherenes

**Figure 1:**
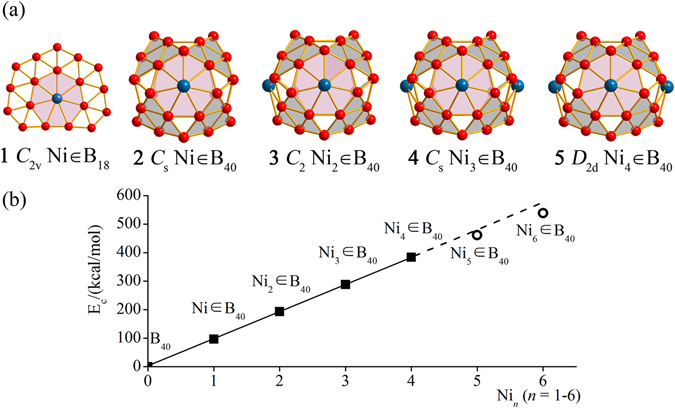
Optimized structures and calculated coordination energies. (**a**) Optimized structures of perfectly planar *C*_2v_ Ni ∈ B_18_ (**1**) and cage-like *C*_s_ Ni ∈ B_40_ (**2**), *C*_2_ Ni_2_ ∈ B_18_ (**3**), *C*_s_ Ni_3_ ∈ B_40_ (**4**), and *D*_2d_ Ni_4_ ∈ B_40_ (**5**) at PBE0/6-311 + G* level. (**b**) Calculated coordination energies (E_c_) of the Ni_n_ ∈ B_40_ heteroborospherenes with respect to Ni_n_B_40_ = Ni_(n-1)_B_40_ + Ni (n = 1–6).The optimized structures of the C_2v_ Ni_5_ ∈ B_40_ (**8**) and D_2d_ Ni_6_ ∈ B_40_ (**9**) are depicted in Fig. S2.

As expected from chemical intuition, the perfectly planar neutral *C*_*2v*_ Ni ∈ B_18_ (**1**) which is isovalent with the experimentally known *C*_*2v*_ Co ∈ B_18_^−^^39^ turns out to be the global minimum of the cluster at CCSD(T) level (Fig. 1 and Fig. S1), with the Ni center and the η^7^-B_7_ heptagon around it matching nicely both geometrically and electronically. The existence of a planar Ni ∈ B_7_ coordination unit in Ni ∈ B_18_ (**1**) presents the possibility of forming Ni-B binary nanostructures with phNi centers in η^7^-B_7_ heptagons. Given the fact that the experimentally observed borospherene *D*_*2d*_ B_40_ possesses four quasi-planar B_7_ heptagons on the waist^22^, it is reasonable to expect that *D*_*2d*_ B_40_ may serve as an effective ligand with four quasi-planar η^7^-B_7_ coordination sites to accommodate phNi centers on the cage surface. It turns out to be true. As indicated in Fig. 1a, Figs S3, and S4, both the cage-like *C*_*s*_ Ni ∈ B_40_ (**2**) and *C*_*2*_ Ni_2_ ∈ B_40_ (**3**) are the global minima of the systems, while *C*_*s*_ Ni_3_ ∈ B_40_ (**4**) and *D*_*2d*_ Ni_4_ ∈ B_40_ (**5**) are true minima. All the phNi centers in the Ni_n_ ∈ B_40_ series (n = 1–4) (**2**–**5**) form effective Ni-B coordination interactions with the η^7^-B_7_ heptagons around them, as indicated by the average Ni-B distance of ${\overline{{\rm{r}}}}_{\mathrm{Ni}-B}$ = 2.02 Å in these clusters which is only slightly longer than the sum (1.95 Å) of the recommended single-bond covalent radii of Ni and B^56^. The twenty low-lying isomers of NiB_40_ all possess cage-like geometries, with the second (*C*_*1*_) and third (*C*_*2v*_) lowest-lying isomers lying about 0.41 eV higher than *C*_*s*_ Ni ∈ B_40_ (**2**) at PBE0 (Fig. S3). The *C*_*2*_ Ni_2_ ∈ B_40_ (**3**) with two phNi centers in two neighboring B_7_ heptagons lies 0.12 eV lower than the second lowest-lying *C*_*2v*_ Ni_2_ ∈ B_40_ which possesses two phNi centers at the opposite sites of the cage (Fig. S4). All the other isomers of Ni_2_B_40_ lie at least 0.42 eV higher than Ni_2_ ∈ B_40_ (**3**) at PBE0. As shown in Fig. S5, the relative stabilities of the most concerned low-lying isomers are found to remain unchanged with Gibbs free energy corrections included at finite temperatures below 600 K. Interestingly, the coordination energies (E_c_) of the Ni_n_ ∈ B_40_ (n = 1–4) series (**2**–**5**) with respect to Ni_n_B_40_ = Ni_(n−1)_B_40_ + Ni (n = 1–4) (referring to triplet Ni and singlet Ni_n_B_40_ in gas phases) increase almost perfectly linearly with the number of Ni atoms in the systems, with the large average E_c_ of 95.90 kcal/mol (4.16 eV/Ni) at PBE0 (Fig. 1b). The E_c_~n linear relation indicates that the four η^7^-B_7_ heptagons in *D*_*2d*_ B_40_ can be practically viewed as four independent coordination sites to accommodate phNi centers. In comparison, the hexacoordinate Ni centers in Ni_5_ ∈ B_40_ (**8**) and Ni_6_ ∈ B_40_ (**9**) at the top and bottom of the B_40_ cage (Fig. S2) possess obviously lower average coordination energies (E_c_ = 75.54 kcal/mol) (Fig. 1b), indicating that phTM centers are favored over hexacoordinate transition-metal centers in the formation of heteroborospherenes. Ni_n_ ∈ B_40_ (n = 1–4) (**2**–**5**) have the large HOMO-LUMO energy gaps of 2.85 eV, 2.59 eV, 2.39 eV, and 2.32 eV at PBE0 level, respectively, well supporting their high chemical stabilities. Similar heteroborospherenes with phPd and phPt centers have also been obtained (Fig. S6).

### Molecular Dynamics Simulations

Extensive molecular dynamics simulations were performed on Ni ∈ B_40_ (**2**) and Ni_2_ ∈ B_40_ (**3**) for 30 ps to allow for crossing of small energy barriers at finite temperatures (Figs S7 and S8). As shown in Figs S7 and S8, both of them are dynamically stable at 500 K, with the root-mean-square-deviation and maximum bond length deviation values of RMSD = 0.07 Å, 0.08 Å and MAXD = 0.25 Å, 0.25 Å, respectively. The two species remain dynamically stable at 700 K, with RMSD = 0.09 Å, 0.09 Å and MAXD = 0.31 Å, 0.31 Å. However, at 1000 K, *C*_*s*_ Ni ∈ B_40_ (**2**) starts to hop between the two lowest-lying *C*_*s*_ and *C*_*1*_ isomers with RMSD = 0.15 Å and MAXD = 0.66 Å, while *C*_*2*_ Ni_2_ ∈ B_40_ (**3**) still keeps its structural integrity.

### Bonding Analyses

**Figure 2:**
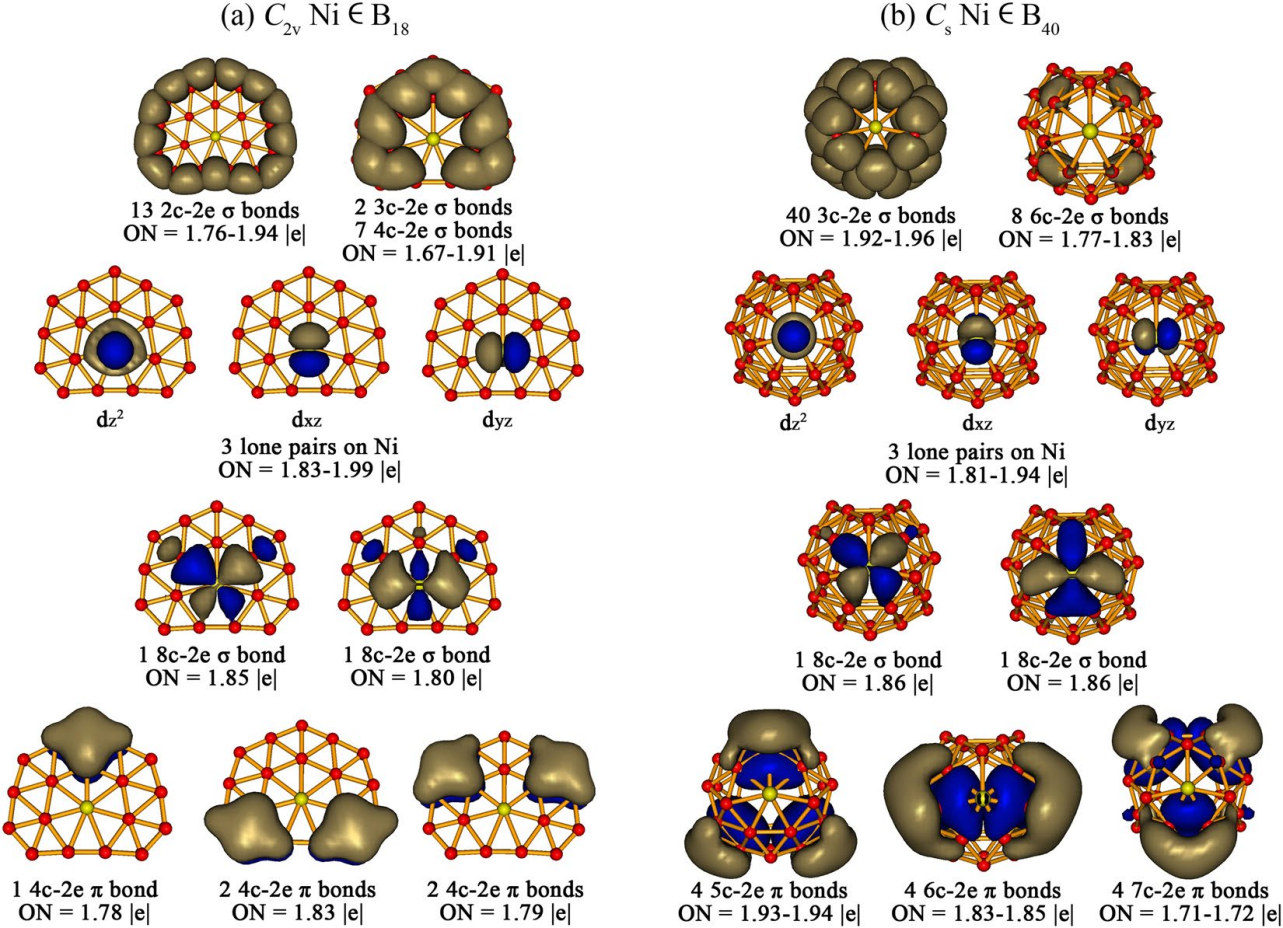
Comparison of the AdNDP bonding patterns of (**a**) *C*_*2v*_ Ni ∈ B_18_ (**1**) and (**b**) *C*_s_ Ni ∈ B_40_ (**2**).

The high stabilities of these heteroborospherenes originate from their electronic structures and bonding patterns. We performed detailed adaptive natural density partitioning (AdNDP)^57, 58^ analyses on the perfectly planar Ni ∈ B_18_ (**1**) and cage-like Ni ∈ B_40_ (**2**) (Fig. 2) which show obvious similarity in bonding patterns though the η^7^-B_7_ heptagon in the latter is slightly off-planed on the cage surface. The phNi center in Ni ∈ B_40_ (**2**) forms three lone pairs in radial direction (*3d*_*z*_^*2*^ *, 3d*_*xz*_^*2*^ *, and 3d*_*yz*_^*2*^) with the occupation numbers of |ON| = 1.81–1.94 |e| and two nearly in-plane 8c-2e σ-coordination bonds between the remaining tangential Ni 3d orbitals (*3*_*x2−y2*_ *and 3d*_*xy*_) and the η^7^-B_7_ heptagon around it with |ON| = 1.86 |e|. The 48 delocalized σ bonds (40 3c-2e and 8 6c-2e) and 12 delocalized π bonds (4 5c-2e, 4 6c-2e, and 4 7c-2e) on the boron framework in Ni ∈B_40_ (**2**) (Fig. 2b) are basically inherited from the parent *D*_*2d*_ B_40_^22^. A similar bonding pattern exists in Ni ∈ B_18_ (**1**) in which the phNi center carries three lone pairs (*3d*_*z*_^*2*^ *, 3d*_*xz*_^*2*^ *, and 3d*_*yz*_^*2*^) in vertical direction with |ON| = 1.83–1.99 |e| and forms two perfectly in-plane 8c-2e σ-coordination bonds with the η^7^-B_7_ heptagon around it with |ON| = 1.80–1.85 |e|, with 22 σ bonds (13 2c-2e, 2 3c-2e, and 7 4c-2e) and 5 4c-2e π bonds on the boron framework outside. Natural bonding orbital (NBO) analyses indicate that the phNi centers in Ni ∈ B_18_ (**1**) and Ni ∈ B_40_ (**2**) possess the electronic configurations of [Ar]*4s* ^*0.00*^ *3d* ^*9.26*^ and [Ar]*4s* ^*0.17*^ *3d* ^*9.34*^, natural atomic charges of + 0.48 |e| and + 0.47 |e|, and total Wiberg bond orders of 1.78 and 1.59, respectively. These results show that the phNi center in **1** and **2** donates its *4 s* ^*2*^ electrons almost completely to the boron framework and, in return, accepts about the same amount of electrons in its partially occupied *3d* tangential orbitals (*3*_*x2−y2*_ and *3d*_*xy*_) from the η^7^-B_7_ heptagon via effective *p* → *d* back-donations. The phNi centers in **1** and **2** have therefore approximately the total bond order of two corresponding to the two 8c-2e σ-coordination bonds between phNi and its η^7^-B_7_ ligand. Such a bonding pattern exists in other phNi-doped Ni_n_ ∈ B_40_ heteroborospherenes (n = 2–4) as well (Figs S9–11). The double delocalization bonding pattern on the B_40_ framework renders 3D aromaticity to these heteroborospherenes, as evidenced by the negative calculated nucleus independent chemical shift (NICS)^59^ values of −43.1, −43.4, −42.4, and −42.2 ppm at the cage centers of Ni ∈ B_40_ (**2**), Ni_2_ ∈ B_40_ (**3**), Ni_3_ ∈ B_40_ (**4**), and Ni_4_ ∈ B_40_ (**5**), respectively, which are well comparable with the corresponding value of −43 ppm calculated for *D*_*2d*_ B_40_^22^.

### IR, Raman, and UV-vis spectra

**Figure 3:**
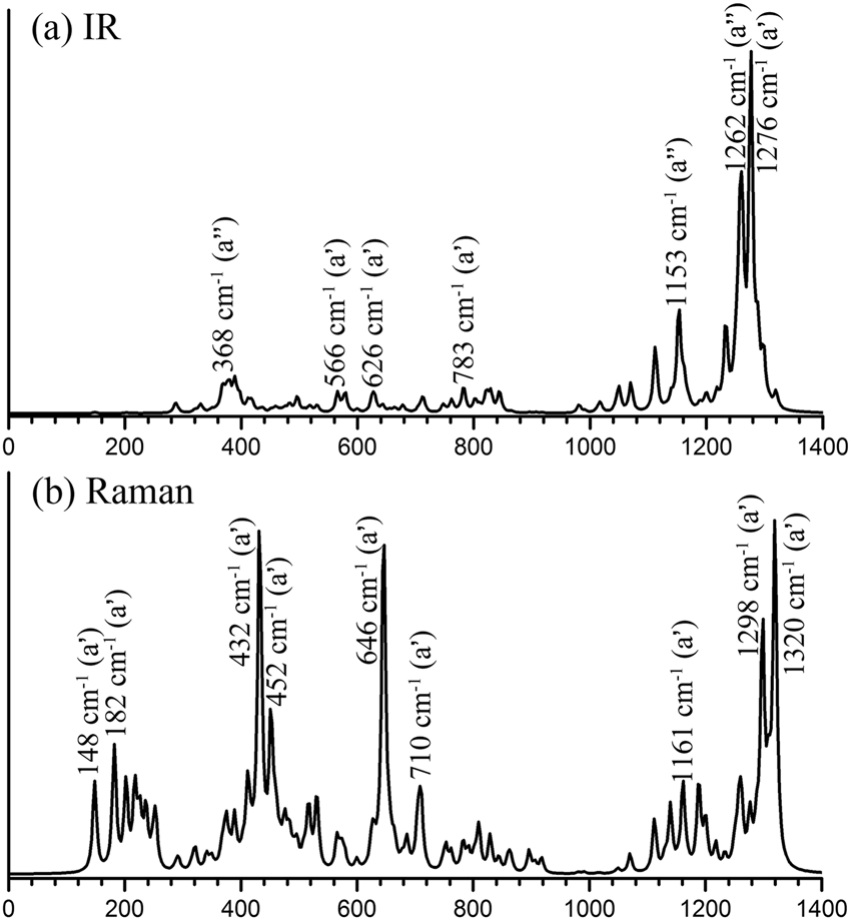
Simulated (**a**) IR and (**b**) Raman spectra of *C*_*s*_ Ni ∈ B_40_ (**2**) at PBE0/6-311 + G* level.

We computationally simulate the IR, Raman, and UV-vis absorption spectra of *C*_*2v*_ Ni ∈ B_18_ (**1**) (Fig. S12) and *C*_*s*_ Ni ∈ B_40_ (**2**) (Fig. 3 and Fig. S13) to facilitate their spectral characterizations. With major IR peaks at 1276 cm^−1^(a’), 1153 cm^−1^ (a”), 783 cm^−1^ (a’), 566 cm^−1^ (a’), and 368 cm^−1^ (a”) and Raman peaks at 1320 cm^−1^ (a’), 1161 cm^−1^ (a’), 646 cm^−1^ (a’), 432 cm^−1^ (a’), 182 cm^−1^ (a’), and 148 cm^−1^ (a’) (Fig. 3), Ni ∈ B_40_ (**2**) exhibits similar spectral features with the parent *D*_*2d*_ B_40_ and *D*_*2d*_ B_40_^+^^24, 25, 60^. These IR and Raman active modes mainly originate from the vibrational transitions of the B_40_ framework. The Raman peaks at 182 cm^−1^ (a’) and 148 cm^−1^ (a’) represent typical radial breathing modes (RBMs) of the *C*_*s*_ Ni ∈ B_40_ (**2**) cage in two perpendicular directions. An intense peak at 210 cm^−1^ was used to characterize the hollow structure of single-walled boron nanotubes^61^. The simulated UV-vis absorption spectroscopy of Ni ∈ B_40_ (**2**) (Fig. S13) using the time-dependent DFT approach^62^ also exhibits certain similarity with that of *D*_*2d*_ B_40_, with the main spectral features located at 201, 217, 242, 275, 301, 324, 415, 501, and 622 nm. Most of the strong absorptions originate from electronic transitions from the deep inner shells to high-lying unoccupied molecular orbitals of the neutral, with the weak absorptions above 500 nm involving transitions from frontier orbitals with Ni *3d* contributions. We also simulated the photoelectron spectra (PES) of the cage-like *C*_*s*_ Ni ∈ B_40_^−^, *C*_*2*_ Ni_2_ ∈ B_40_^−^, *C*_*s*_ Ni_3_ ∈ B_40_^−^, and *D*_*2d*_ Ni_4_ ∈ B_40_^−^ monoanions (Fig. S14) which appear to have the low calculated first vertical and adiabatic detachment energies of VDE/ADE = 2.40/2.30, 2.39/2.29, 2.40/2.32, and 2.34/2.26 eV, respectively, followed by a sizable energy gap of about 1.0 eV, similar to the measured PES of the cage-like *D*_*2d*_ B_40_^−^^22^.

### Heteroborophene monolayers Ni_2_ ∈ B_14_ (6, 7)

**Figure 4:**
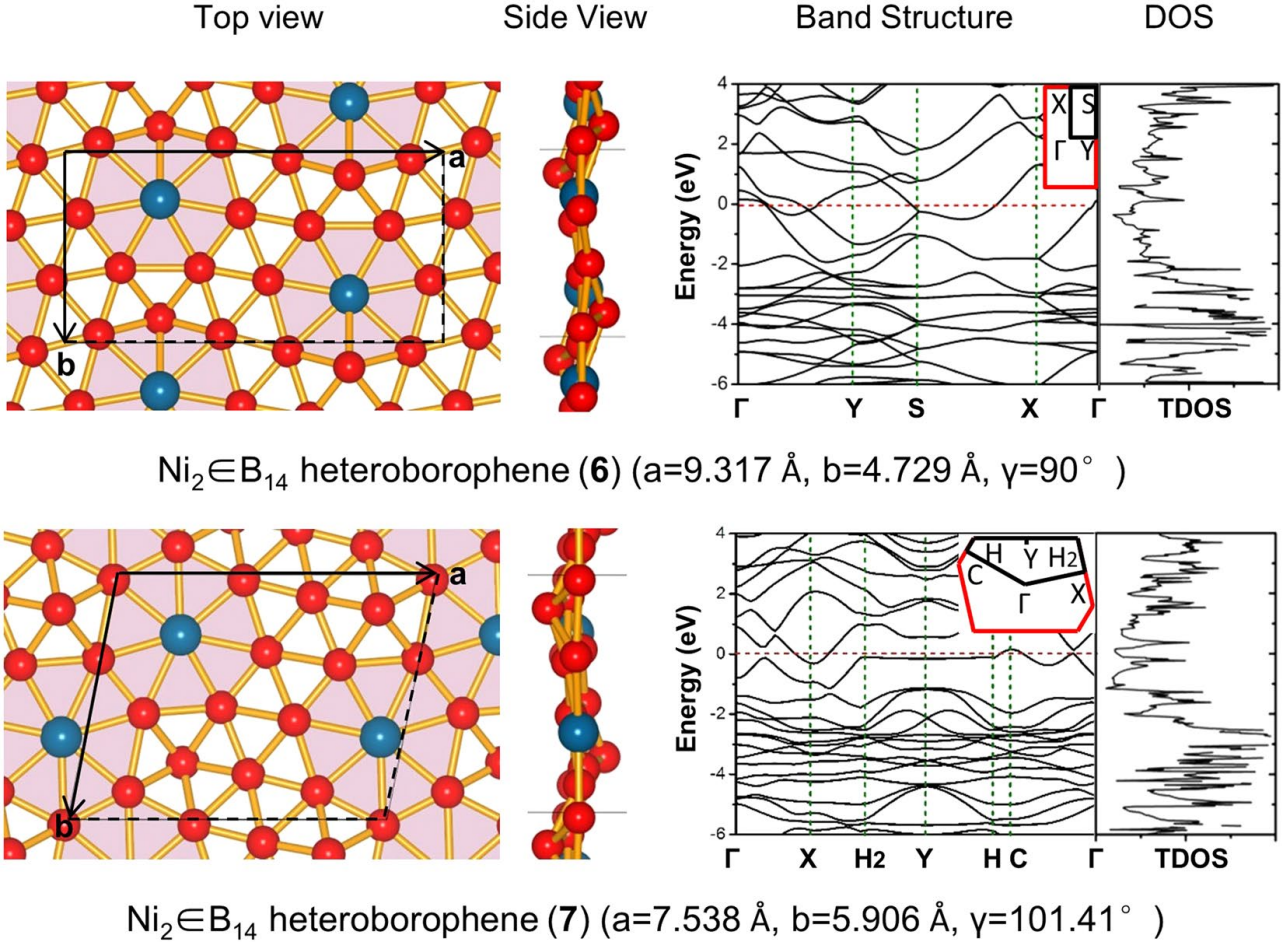
Top and side views of the structures of 2D heteroborophenes. Ni_2_ ∈ B_14_ (**6**) and Ni_2_ ∈ B_14_ (**7**) were optimized at PBE and their band structures and total densities of states (TDOS) were calculated at HSE06. The insets represent the shapes of first Brillouin zones. Γ, X, A and Z of (**6**) correspond to the (0, 0, 0), (0, 0.5, 0), (0.5, 0.5, 0) and (0.5, 0, 0) k-points, while Γ, X, H_2_, Y, H and C of (**7**) correspond to the (0, 0, 0), (0.5, 0, 0), (0.440, 0.389, 0), (0, 0.5, 0), (−0.440, 0.611, 0) and (−0.5, 0.5, 0) k-points.

To locate the planar monolayer precursor of the cage-like heteroborospherenes (**2**–**5)**, we performed an extensive GM search on a Ni_2_B_14_ monolayer which contains a unit cell doubling the size of a planar NiB_7_ coordination unit. With more than 950 valid structures probed, the nearly degenerate Ni_2_ ∈ B_14_ (**6**) (*P2*_*1*_ */m*) and Ni_2_ ∈ B_14_ (**7**) (*P-1*) monolayers (Fig. 4) which may coexist in experiments turn out to be the two lowest-lying 2D polymorphs of the system (Fig. S15) without imaginary phonon dispersion frequencies at PBE level^54^ (Fig. S16). Slight buckling occurs in these heteroborophene monolayers with the maximum buckled heights of 1.16 Å in **6** and 1.51 Å in **7** at the pentacoordinate B sites, similar to the symmetric buckling observed in silicene^63^. Ni_2_ ∈ B_14_ (**6**) and Ni_2_ ∈ B_14_ (**7**) possess the average cohesive energies of *E*_coh_ = 5.932 eV and 5.939 eV per atom (*E*_coh_ = (2*E*_Ni_ + 14*E*_B_ − *E*_Ni2B14_)/16), respectively, which are higher than the cohesive energies of the recently proposed FeB_6_ (5.79 eV/atom) and FeB_2_ (4.87 eV/atom) at the same theoretical level^37, 38^. The current prediction of heteroborophene monolayers **6** and **7** with nearly in-plane phNi centers is supported by the recently observed perfectly planar heteroborophene-type Co ∈ B_18_^−^ cluster which contains a phCo center^39^. More intriguingly, the Ni_2_ ∈ B_14_ (**6**) monolayer composed of interwoven BDCs with two equivalent phNi centers per unit cell turns out to be the precursor of the cage-like heteroborospherenes (**2**–**5**) which, with four heptagons on the waist and two hexagons at the top and bottom, can be unfolded into Ni_2_ ∈ B_14_ (**6**) monolayer by repetition simultaneously along the orthogonal direction (Fig. S17). This observation builds an interesting link between heteroborophene monolayers and cage-like heteroborospherenes. Lying 0.007 eV/atom higher in cohesive energy than Ni_2_ ∈ B_14_ (**6**), Ni_2_ ∈ B_14_ (**7**) with both capped pentagons (B_6_) and hexagons (B_7_) also contains two equivalent phNi centers per unit cell. Both Ni_2_ ∈ B_14_ (**6**) and Ni_2_ ∈ B_14_ (**7**) monolayers turn out to be metallic in nature, as shown in their calculated band structures and total densities of states (Fig. 4) which possess non-zero densities at Fermi levels, similar to boron α-sheet and other low-lying boron monolayers^34–36^. Detailed SSAdNDP bonding analyses^57, 58^ (Fig. S18) show that both Ni_2_ ∈ B_14_ (**6**) and Ni_2_ ∈ B_14_ (**7**) possess a σ + π double delocalization bonding pattern on their boron frameworks, with each phNi center forming three lone pairs (*3d*_*z*_^2^ *, 3d*_*xz*_^*2*^ *, and 3d*_*yz*_^*2*^) in vertical direction and two nearly in-plane 8c-2e σ-coordination bonds with the η^7^-B_7_ pentagon around it. Such a bonding pattern is well in line with the bonding patterns in both planar Ni ∈ B_18_ (**1**) and cage-like Ni_n_ ∈ B_40_ (n = 1–4) (**2**–**5**) discussed above. The σ + π double delocalization bonding pattern on the boron frameworks renders metallicity to these 2D heteroborophenes.

In summary, based on extensive first-principles calculations, we have presented the possibility of the novel planar heteroborophene-type cluster Ni ∈ B_18_ (**1**), 3D aromatic heteroborospherenes Ni_n_ ∈ B_40_ (n = 1–4) (**2**–**5**), and 2D metallic heteroborophenes Ni_2_ ∈ B_14_ (**6, 7**) which all contain phNi centers in η^7^-B_7_ pentagons with the universal bonding pattern of σ + π double delocalization on their boron frameworks, with each phNi forming two effective 8c-2e σ-coordination bonds with the η^7^-B_7_ heptagon around it. Initial investigations suggest that phNi centers in **2**–**7** can be substituted by phPd, phPt, or other phTM dopants with the right atomic radii and electronic configurations to form stable heteroborospherenes, heteroborophenes, and heteroboronanotubes. Possible free-standing heteroboronanostructures stabilized by phTM centers guarantee further experimental and theoretical investigations to expand the chemistry and materials science of boron which are expected to be complementary to that of carbon^31–38^.

## Electronic supplementary material

Supplementary Information 
