# Supplementary material for: Heteroborospherene clusters Nin ∈ B40 (n = 1–4) and heteroborophene monolayers Ni2 ∈ B14 with planar heptacoordinate transition-metal centers in η7-B7 heptagons
Source: Sci Rep. 2017 Jul 18;7:5701. doi: 10.1038/s41598-017-06039-9 (PMC5515878; doi:10.1038/s41598-017-06039-9)
Supplement: Supplementary file 1 — Supplementary Information [file 41598_2017_6039_MOESM1_ESM.pdf]

Supporting Information

Heteroborospherene clusters $Ni_n \in B_{40}$ (n=1-4) and heteroborophene monolayers $Ni_2 \in B_{14}$ with planar heptacoordinate transition-metal centers in η^7-B_7 heptagons

Hai-Ru Li, Xin-Xin Tian, Xue-Mei Luo, Miao Yan, Yue-Wen Mu, Hai-Gang Lu, and Si-Dian Li

Contents

Methods Section

Figure S1. Optimized low-lying isomers of NiB_{18}.

Figure S2. Optimized structures of C_{2v} $Ni_5\in B_{40}$ and D_{2d} $Ni_6\in B_{40}$.

Figure S3. Optimized low-lying isomers of NiB_{40}.

Figure S4. Optimized low-lying isomers of Ni_2B_{40}.

Figure S5. Relative Gibbs free energies of NiB_{18} and NiB_{40}.

Figure S6. Optimized structures of $Pd_n\in B_{40}$ and $Pt_n\in B_{40}$ (n=1-4).

Figure S7. BOMD of C_s Ni∈B40.

Figure S8. BOMD of C_2 $Ni_2\in$B40.

Figure S9. AdNDP analyses of C_2 $Ni_2\in B_{40}$.

Figure S10. AdNDP analyses of C_s $Ni_3\in B_{40}$.

Figure S11. AdNDP analyses of D_{2d} $Ni_2\in B_{40}$.

Figure S12. Simulated IR, Raman and UV-vis spectra of C_{2v} $Ni\in B_{18}$.

Figure S13. Simulated UV-vis spectrum of C_s $Ni\in B_{40}$.

Figure S14. Simulated PES spectra of $Ni_n\in B_{40}^-$ (n=1-4).

Figure S15. Optimized low-lying isomers of Ni_2B_{14}.

Figure S16. The phonon dispersion curves of $Ni_2\in B_{14}$.

Figure S17. Heterborospherenes $Ni_n\in B_{40}$ (n=1-4) and their precursor heteroborophene monolayer $Ni_2\in B_{14}$.

Figure S18. SSAdNDP analyses of $Ni_2\in B_{14}$ (**6** and **7**).

Table S1. Optimized coordinates of **1-7**.

Methods Section:

GM searches for NiB_{18}, NiB_{40} and Ni_2B_{40} were performed using the minima hopping (MH) algorithm[1,2] and TGmin code[3] developed based on the Basin Hopping (BH) algorithm[4] at the DFT level, combined with manual structural constructions based on the known planar, tubular, and cage-like boron clusters. The TGmin program developed at Tsinghua University was employed to search for the global minimum (GM) and used to search for the GM previously. About 1500, 1734, and 1700 stationary points on the potential surfaces were probed for NiB_{18}, NiB_{40}, and Ni_2B_{40}. Low-lying isomers were optimized at the PBE0[5] and TPSSh[6] levels with the 6-311+G*[7] basis set for B and Ni and Stuttgart relativistic small-core pseudopotential and valence basis set for Pd and Pt.[8,9] All the energies were corrected for zero-point energies. Frequency calculations were done to ensure that the isomers are true minima. The top five lowest isomers single-point energy calculations were refined using the coupled cluster method with triple excitations (CCSD(T))[10-12] implemented in MOLPRO[13] with the 6-31G* basis set at PBE0/6-311+G* geometries for NiB_{18} and NiB_{40}. The simulated photoelectron spectra (PES) were calculated using time-dependent DFT (TD-DFT)[14]. The first vertical detachment energy (VDE) for Ni_nB_{40} (n=1-4) was calculated by the energy difference between the anion and the neutral at the respective anion geometry. Natural atomic charges were analyzed using the NBO 5.0 program[15]. Born-Oppenheimer molecular dynamics (MD) simulations were performed via the software suite CP2K[16]. Chemical bonding analyses were performed using the adaptive natural density partitioning (AdNDP)[17] method at the PBE0/6-31G level and Solid State Adaptive Natural Density Partitioning (SSAdNDP)[18] procedure for NiB_{18}, Ni_nB_{40} (n = 1-4) and Ni_2B_{14}, respectively, and visualized using Molekel[19] program and Visualization for Electronic STructural Analyses (VESTA)[20]. All clusters electronic structure calculations were performed using Gaussian 09 package[21].

A general global search method based on the PSO technique implemented in the Crystal Structure Analysis by Particle Swarm Optimization (CALYPSO) package was employed[22]. The underlying plane-wave based density functional theory calculations were performed by using the Vienna *ab initio* simulation package (VASP)[23,24], within the framework of projector augmented wave (PAW) pseudopotential method[25,26] and PBE generalized gradient approximation (GGA)[27]. For global search simulations, the cutoff energy was set to 400 eV, the Brillouin zones were sampled with 0.5 $Å^{-1}$ spacing in reciprocal space by the Monkhorst-Pack scheme[28], and the vacuum gap between two slabs was set to 15 Å; geometry optimization was done when the atomic force was less than 0.01 eV/Å and the energy difference was lower than 10^{-4} eV. To determine the Global Minimum, cutoff energy of 500 eV and energy tolerance of 10^{-5} eV was set for further optimization and energy tolerance of 10^{-6} eV was set for single point calculation. The band structure of Ni_2B_{14} sheet was also calculated using the VASP code. The phonon

spectrum was calculated by finite displacement method implemented in Phonopy program[29]. The Heyd−Scuseria−Ernzerhof (HSE06) approach[30] was used to calculate the band structures and densities of states of the PBE structures.

References

1. Goedecker, S. Minima hopping: an efficient search method for the global minimum of the potential energy surface of complex molecular systems. *J. Chem. Phys.* **120**, 9911─9917 (2004).
2. Goedecker, S., Hellmann, W. & Lenosky, T. Global minimum determination of the Born-Oppenheimer surface within density functional theory. *Phys. Rev. Lett.* **95**, 055501 (2005).
3. Chen, X., Zhao, Y. F., Wang, L.-S. & Li, J. Recent progresses of global minimum search of nanoclusters with a constrained Basin-Hopping algorithm in the TGmin program. *Comput. Theor. Chem.* **1107**, 57-65 (2017).
4. Wales, D. J. & Scheraga, H. A. Global optimization of clusters, crystals, and biomolecules. *Science* **285**, 1368─1372 (1999).
5. Adamo, C. & Barone, V. Toward reliable density functional methods without adjustable parameters: the PBE0 model. *J. Chem. Phys.* **110**, 6158─6170 (1999).
6. Tao, J., Perdew, J. P., Staroverov, V. N. & Scuseria, G. E. Climbing the density functional ladder: nonempirical meta-generalized gradient approximation designed for molecules and solids. *Phys. Rev. Lett.* **91**, 146401 (2013).
7. Krishnan, R., Binkley, J. S., Seeger, R. & Pople, J. A. Self-consistent molecular orbital methods. XX. A basis set for correlated wave functions. *J. Chem. Phys.* **72**, 650─654 (1980).
8. Feller, D. The role of databases in support of computational chemistry calculations. *J. Comput. Chem.* **17**, 1571 – 1586 (1996).
9. Schuchardt, K. L. *et al*. *J. Chem. Inf. Model.* **47**, 1045 – 1052 (2007).
10. Čížek, J. On the use of the cluster expansion and the technique of diagrams in calculations of correlation effects in atoms and molecules. *Adv. Chem. Phys.* **14**, 35–89 (1969).
11. Purvis, G. D. & Bartlett, R. J. A full coupled-cluster singles and doubles model: The inclusion of disconnected triples. *J. Chem. Phys.* **76**, 1910–1918 (1982).

12. Raghavachari, K., Trucks, G. W., Pople, J. A. & Head-Gordon, M. A fifth-order perturbation comparison of electron correlation theories. *Chem. Phys. Lett.* **157**, 479–483 (1989).
13. Werner, H. J. *et al*. *Molpro*, version 2012.1 (www.molpro.net).
14. Bauernschmitt, R., Ahlrichs, R., Treatment of electronic excitations within the adiabatic approximation of time dependent density functional theory. *Chem. Phy. Lett.* **256**, 454 (1996).
15. Glendening, E. D. *et al.* NBO 5.0, Theoretical Chemistry Institute, University of Wisconsin, Madison (2001**)**.
16. VandeVondele, J. *et al*. Quickstep: fast and accurate density functional calculations using a mixed Gaussian and plane waves approach. *Comput. Phys. Commun.* **167**, 103–108 (2005).
17. Zubarev, D. Y. & Boldyrev, A. I. Developing paradigms of chemical bonding: adaptive natural density partitioning. *Phys. Chem. Chem. Phys.* **10**, 5207–5217(2008).
18. Galeev, T. R., Dunnington, B. D., Schmidt, J. R. & Boldyrev, A. I. Solid state adaptive natural density partitioning: a tool for deciphering multi-center bonding in periodic systems. *Phys. Chem. Chem. Phys.* **15**, 5022–5029 (2013).
19. Varetto, U. Molekel 5.4.0.8, Swiss National Supercomputing Centre, Manno, Switzerland, (2009).
20. Momma, K. & Izumi, F. VESTA 3 for three-dimensional of crystal, volumetric and morphology data. *J. Appl. Catal.* **44**, 1272–1376 (2011).
21. Frisch, M. J. *et al.* Gaussian Inc., Wallingford, CT, (2009).
22. Wang, Y.-C., Lv, J., Zhu, L. & Ma, Y.-M. Crystal structure prediction via particle-swarm optimization. *Phys. Rev. B* **82**, 094116 (2010).
23. Kresse, G. & Furthmuller, J. Efficient iterative schemes for *ab initio* total-energy calculations using a plane-wave basis set. *J. Phys. Rev. B* **54**, 11169–11186 (1996).
24. Kresse, G. & Hafner, J. Norm-conserving and ultrasoft pseudopotentials for first-row and transition elements. *J. Phys. Condens. Matter* **6**, 8245–8257 (1994).
25. Blochl, P. E. Projector augmented-rave method. *Phys. Rev. B* **50**, 17953–17979 (1994).
26. Kresse, G. & Joubert, D. From ultrasoft pseudopotentials to the projector augmented-wave method. *Phys. Rev. B* **59**, 1758–1775 (1999).
27. Perdew, J. P., Burke, K. & Ernzerhof, M. Generalized gradient approximation made simple. *Phys. Rev. Lett.* **77**, 3865–3868 (1996).
28. Monkhorst, H. J. & Pack, J. D. Special points for Brillouin-zone integrations. *Phys. Rev. B* **13**, 5188–5192 (1976).

29. Togo, A., Oba, F. & Tanaka, I. First-principles calculation of the ferroelastic transition between rutile-type and $CaCl_2$-type SiO_2 at high pressures. *Phys. Rev. B 78*, 134106 (2008).

30. Heyd, J., Scuseria, G. E. & Ernzerhof, M. Hybrid functionals based on a screened coulomb potential. *J. Chem. Phys.* **118**, 8207–8215 (2003).

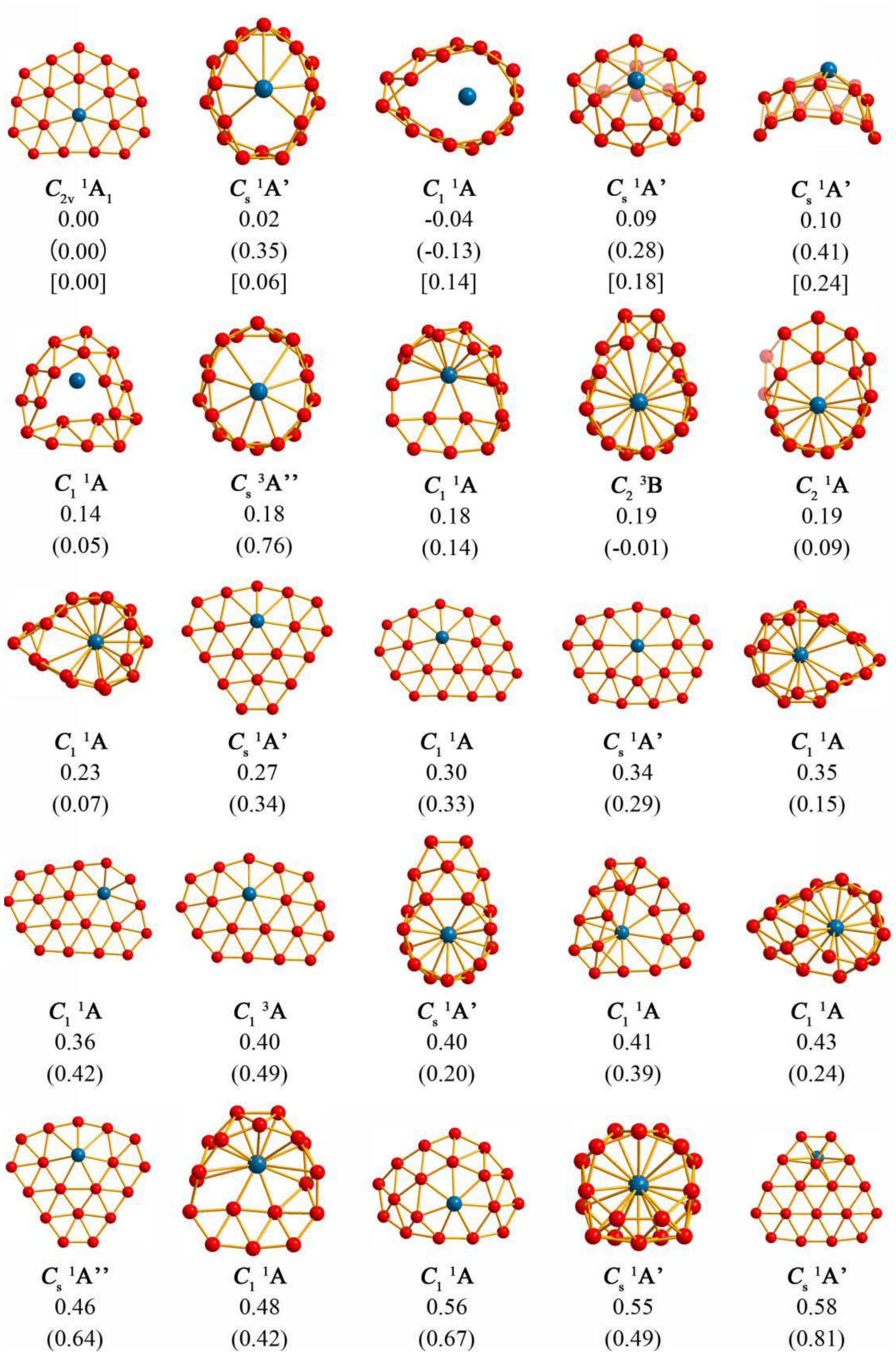

Figure S1. Low-lying isomers of NiB_{18} with their relative energies (with zero-point corrections included)

indicated in eV at the PBE0, TPSSh (in parenthesis), and CCSD(T) (in square brackets) levels.

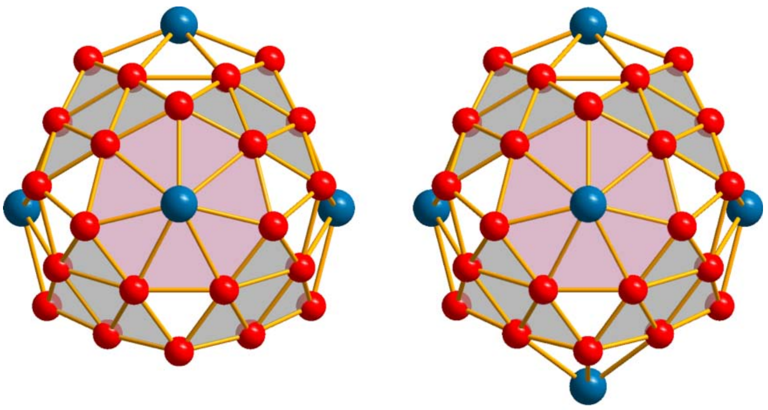

Figure S2. The optimized structures of C_{2v} $Ni_5 \in B_{40}$ (**8**) and D_{2d} $Ni_6 \in B_{40}$ (**9**).

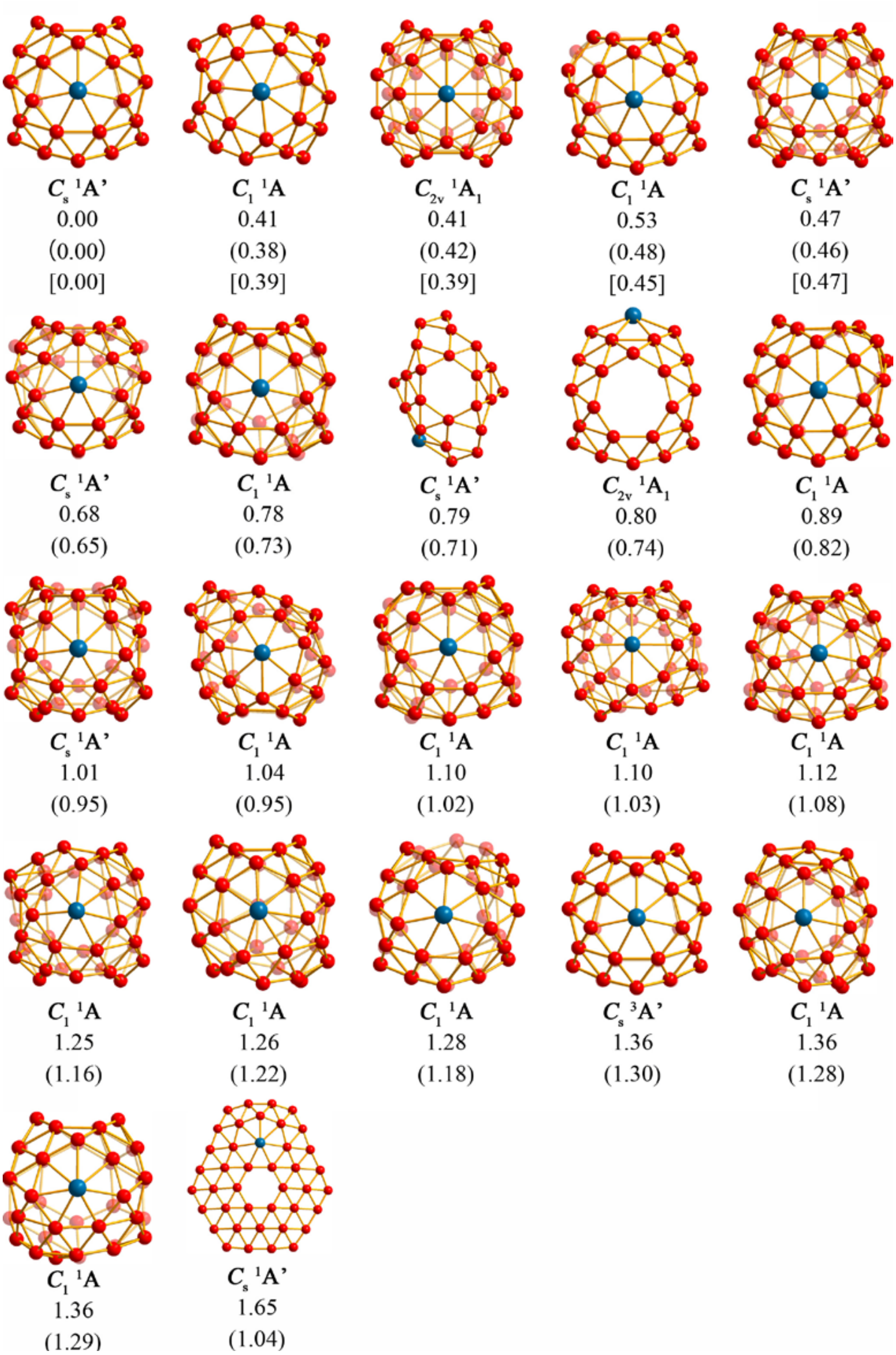

Figure S3. Low-lying isomers of NiB_{40} with their relative energies (with zero-point corrections included) indicated in eV at the PBE0, TPSSh (in parenthesis), and CCSD(T) (in square brackets) levels.

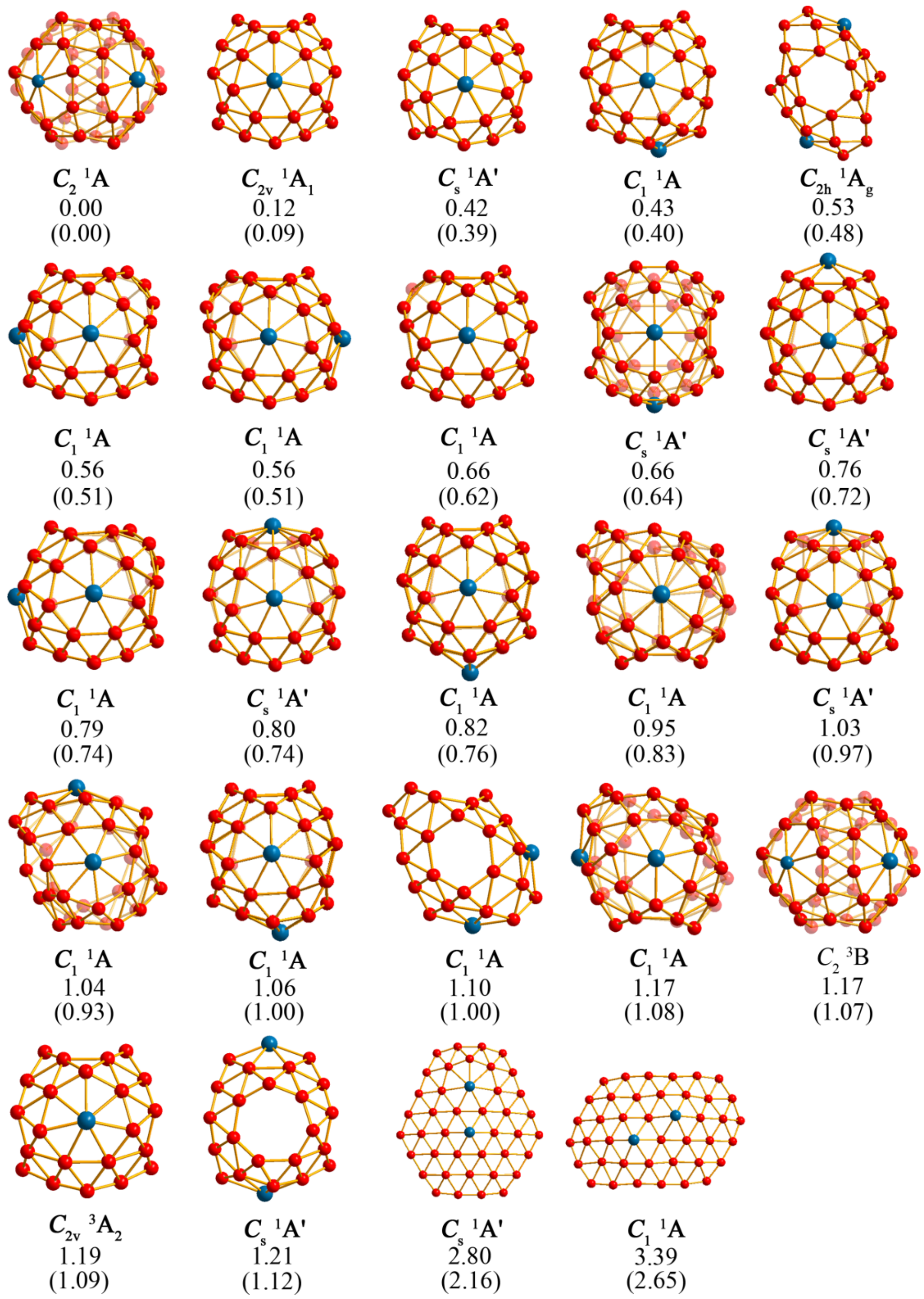

Figure S4. Low-lying isomers of Ni_2B_{40} with their relative energies (with zero-point corrections included) indicated in eV at the PBE0 and TPSSh (in parenthesis) levels.

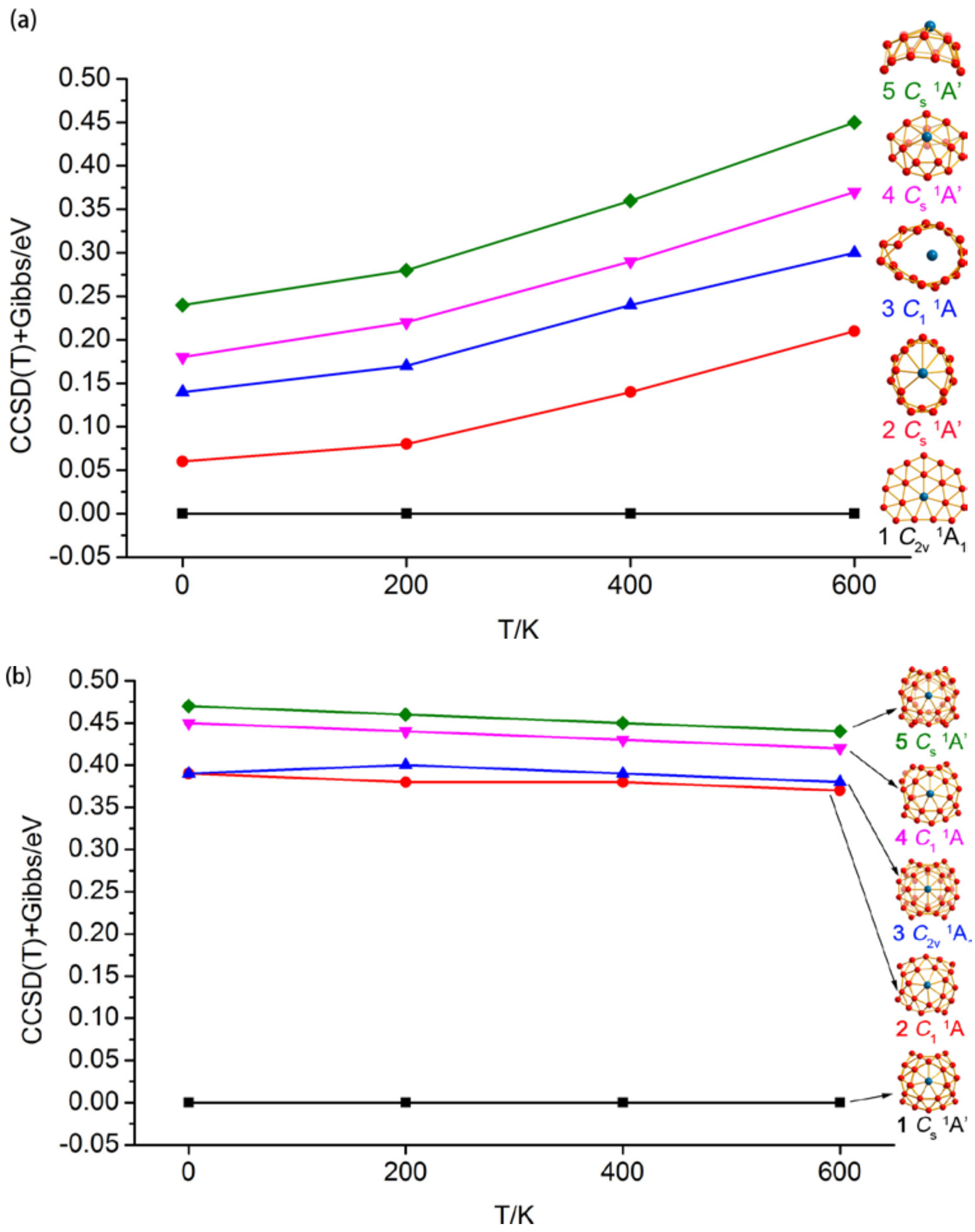

Figure S5. Relative energies of the top five isomers of (a) NiB_{18} and (b) NiB_{40} at the single-point CCSD(T) level, with Gibbs free energy corrections included at PBE0 level as a function of temperature at 0, 200, 400 and 600K. The energies are plotted relative to that of the GM C_{2v} NiB_{18} and C_s NiB_{40} structures, respectively.

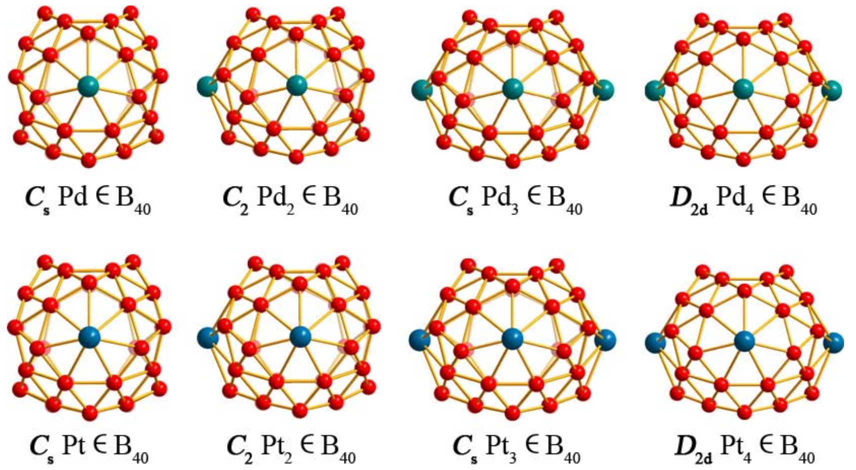

Figure S6. The heteroborospherenes structures of phPd and phPt at the PBE0 level.

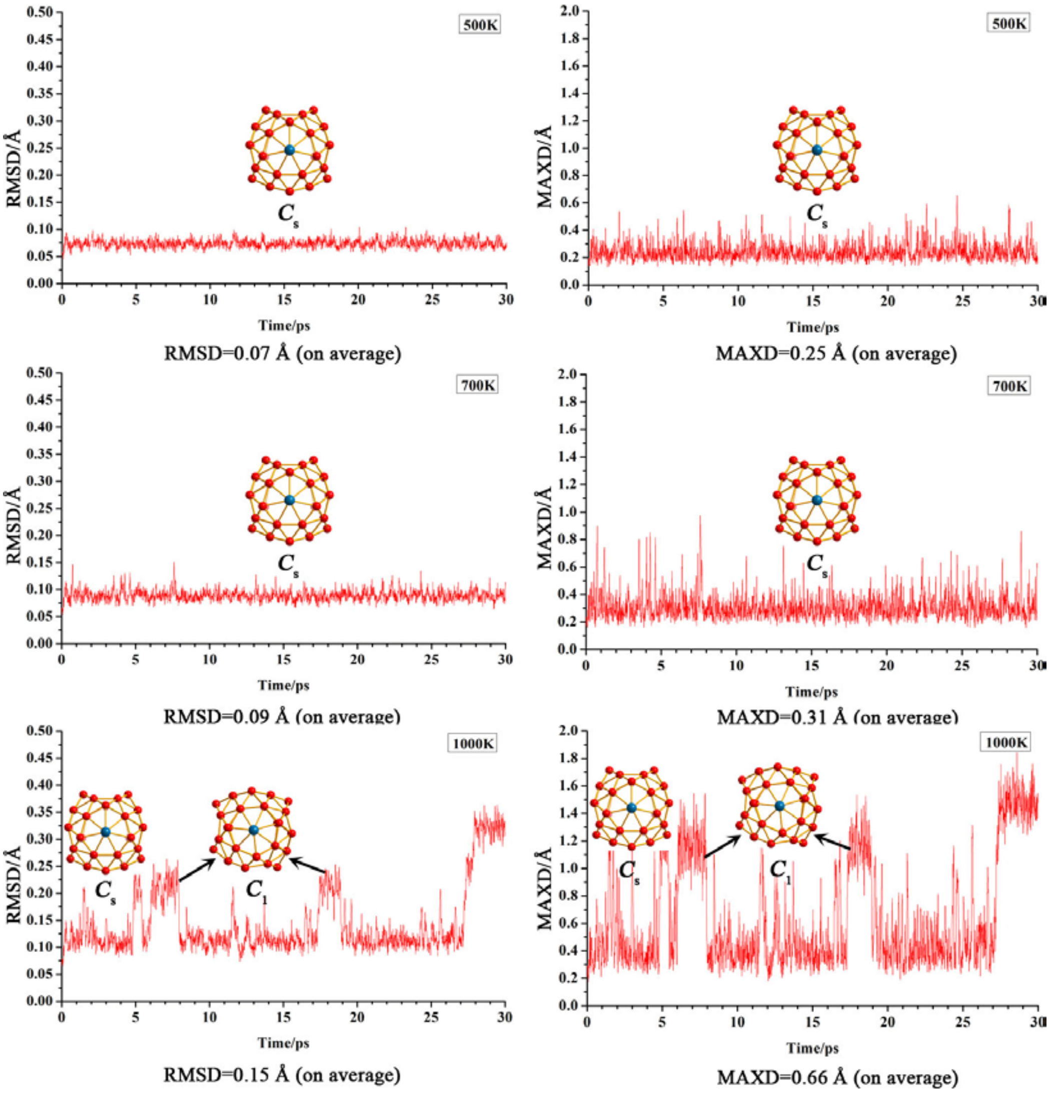

Figure S7. Born-Oppenheimer molecular dynamics (BOMD) simulations of C_s Ni∈B_{40} at 500K, 700K and 1000K for 30ps, respectively. The root-mean-square-deviation (RMSD) and maximum bond length deviation (MAXD) values are displayed in Å (on average).

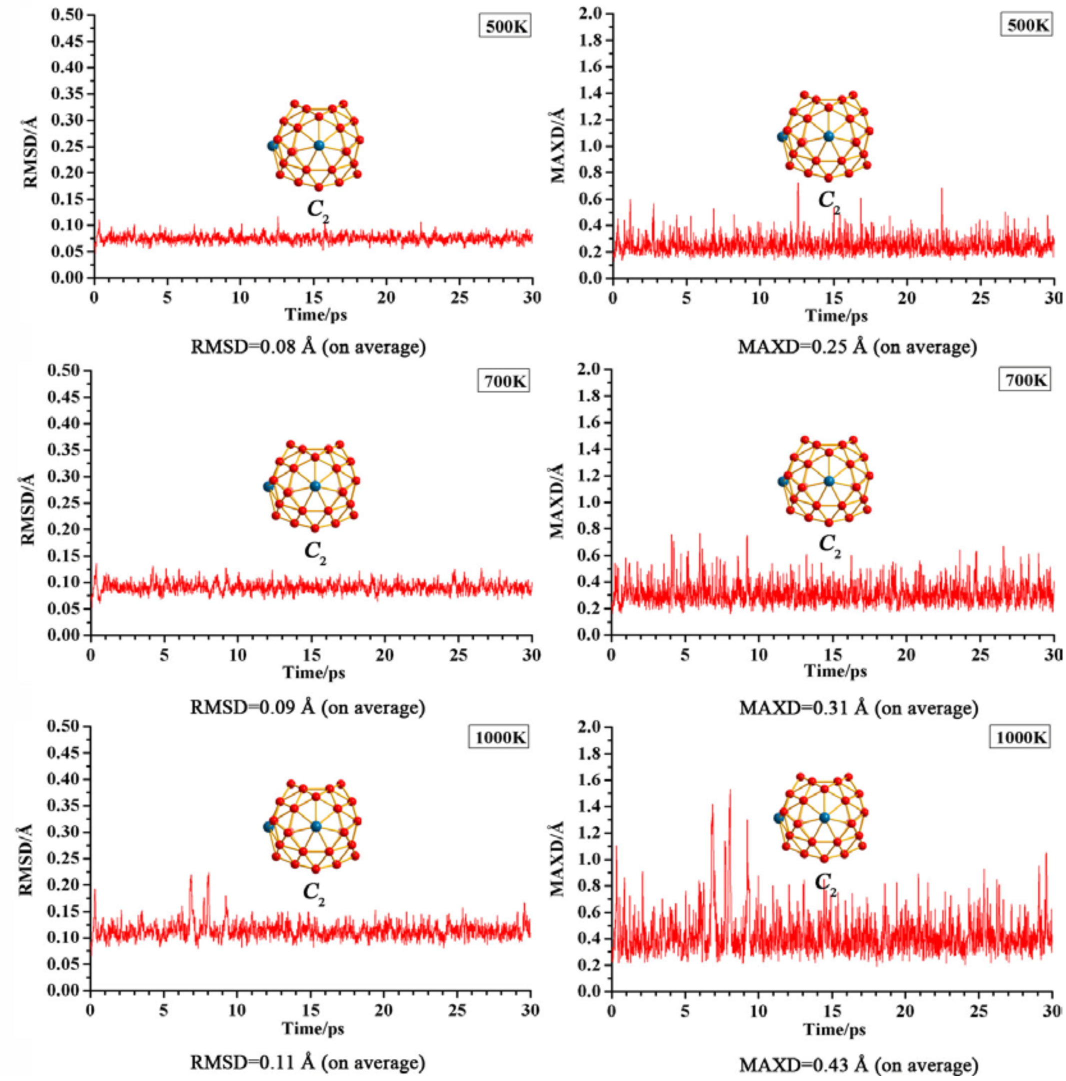

Figure S8. Born-Oppenheimer molecular dynamics simulations (BOMD) of C_2 $Ni_2{\in}B_{40}$ at 500K, 700K and 1000K for 30ps, respectively. The root-mean-square-deviation (RMSD) and maximum bond length deviation (MAXD) values are displayed in Å (on average).

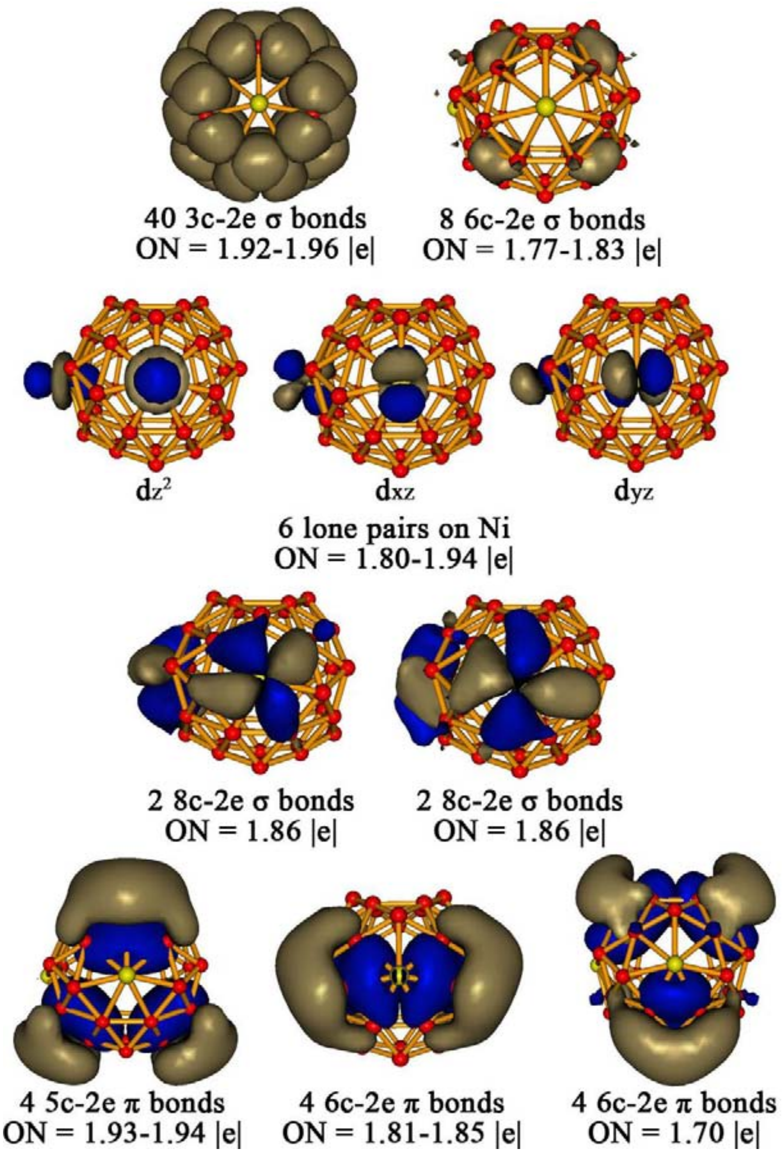

Figure S9. AdNDP bonding patterns of C_2 $Ni_2{\in}B_{40}$.

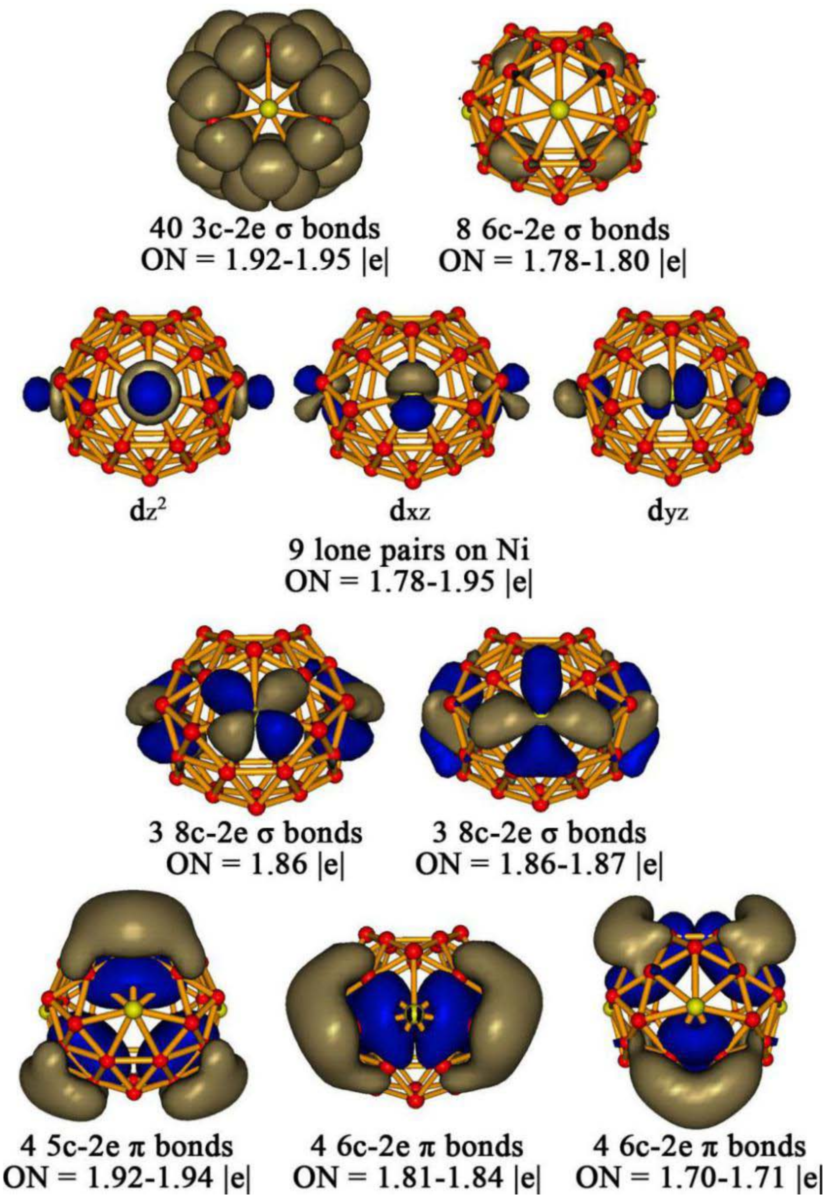

Figure S10. AdNDP bonding patterns of C_s $Ni_3{\in}B_{40}$.

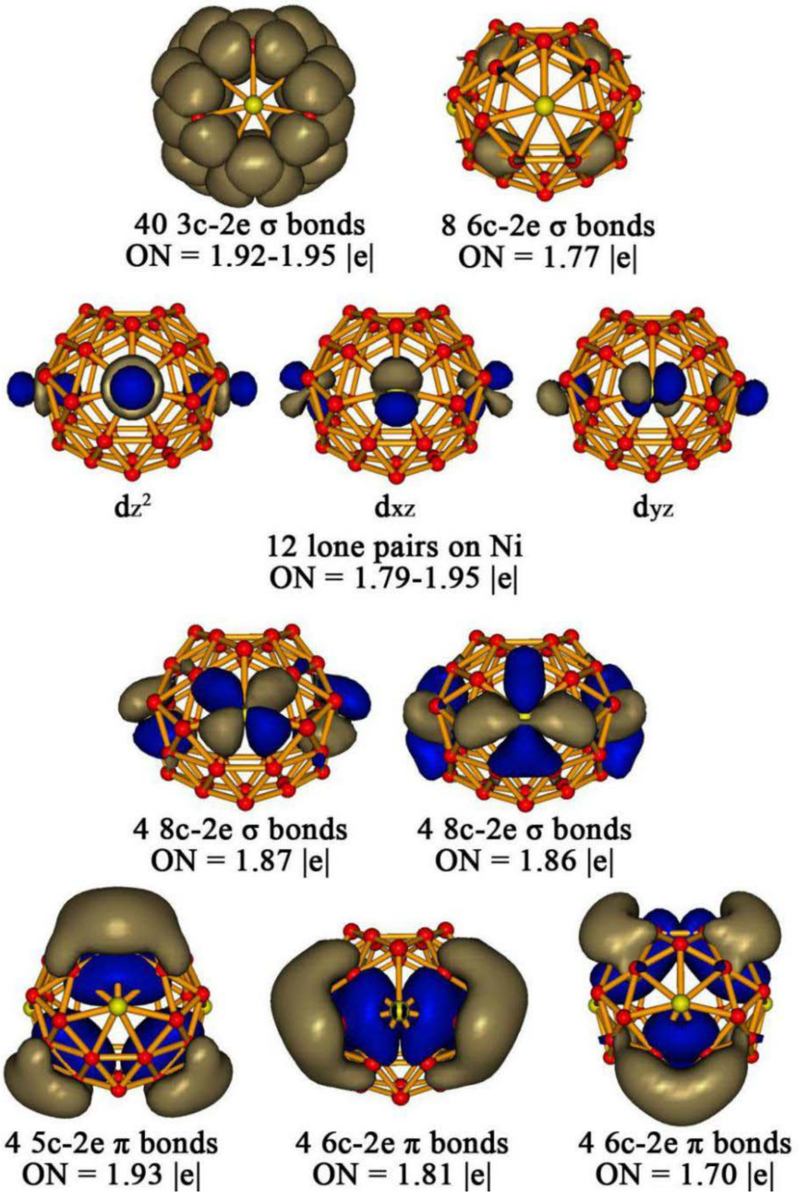

Figure S11. AdNDP bonding patterns of D_{2d} $Ni_4 \in B_{40}$.

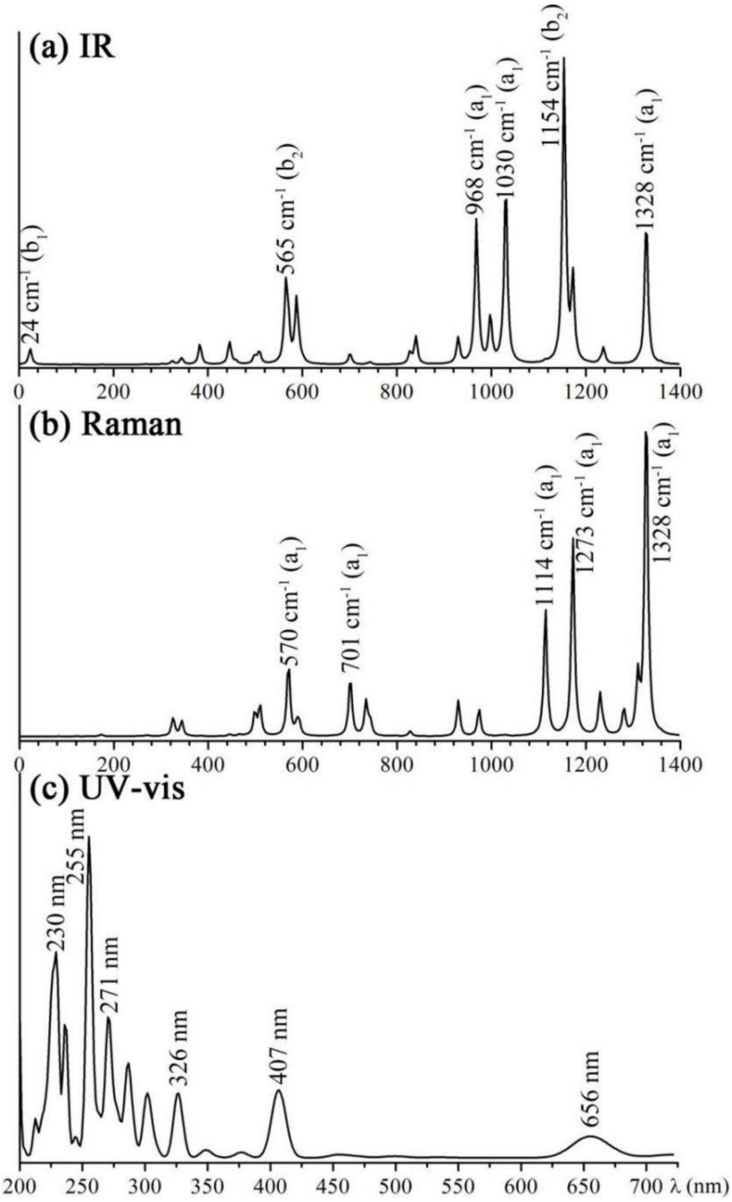

Figure S12. Simulated the IR, Raman and UV-vis spectra of C_{2v} Ni∈B_{18} (**1**) at the PBE0 level.

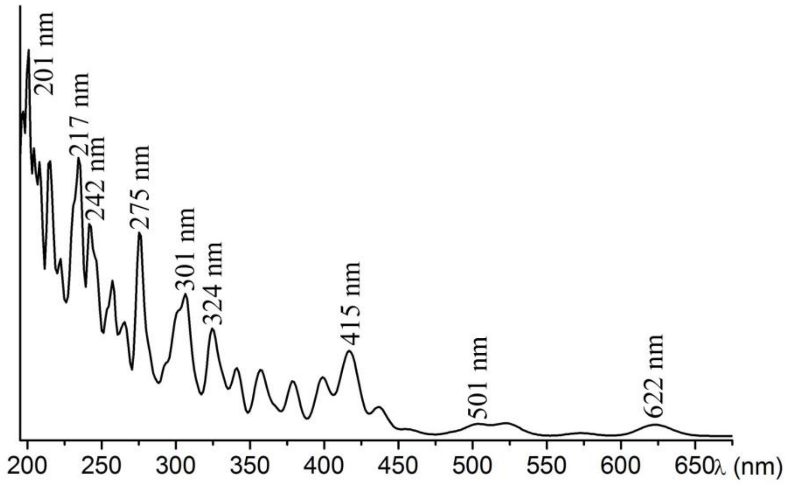

Figure S13. Simulated UV-vis absorption spectrum of C_s Ni∈B_{40} (**2**) at the PBE0 level.

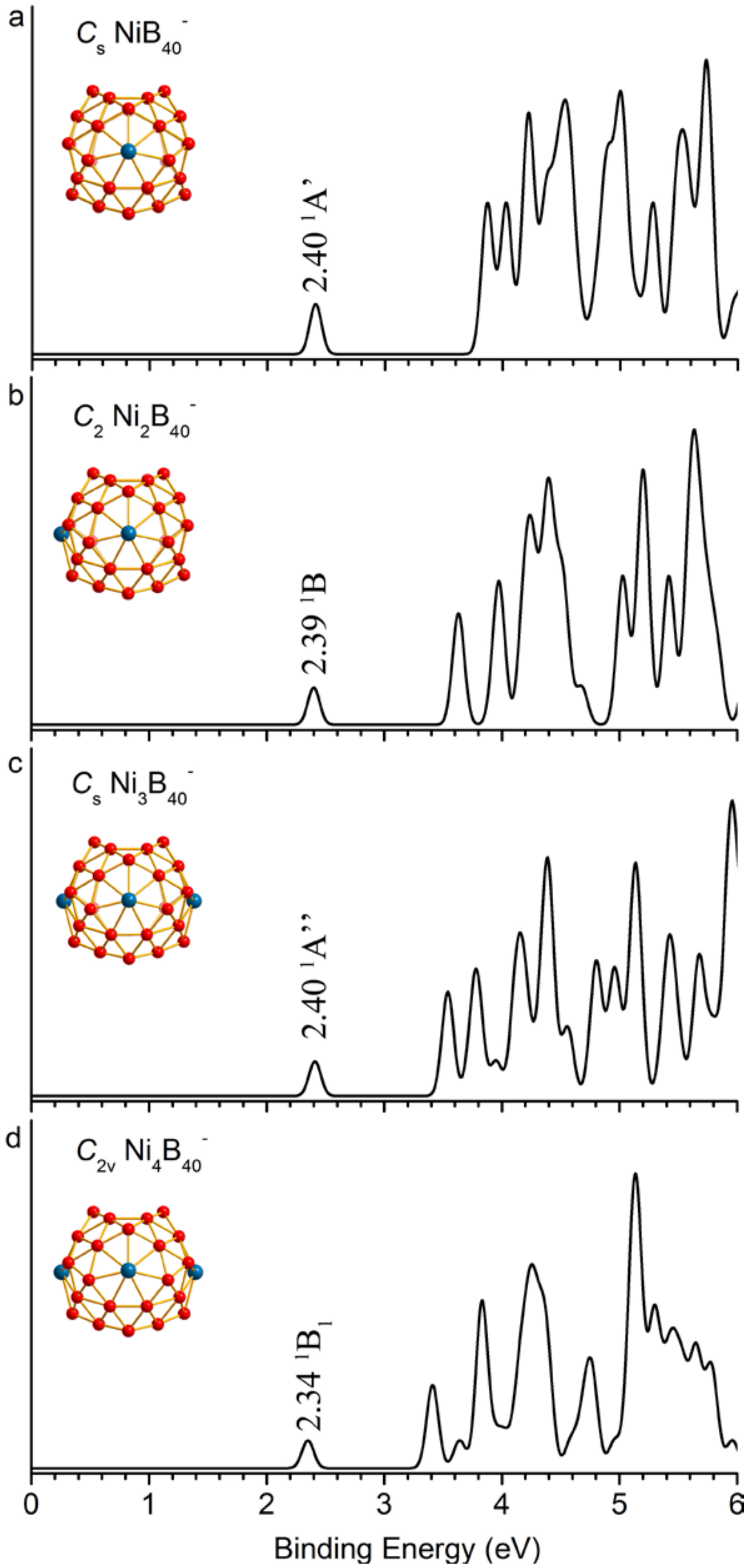

Figure S14. Simulated PES spectra of $Ni_n \in B_{40}^-$ (n=1-4) at the PBE0 level.

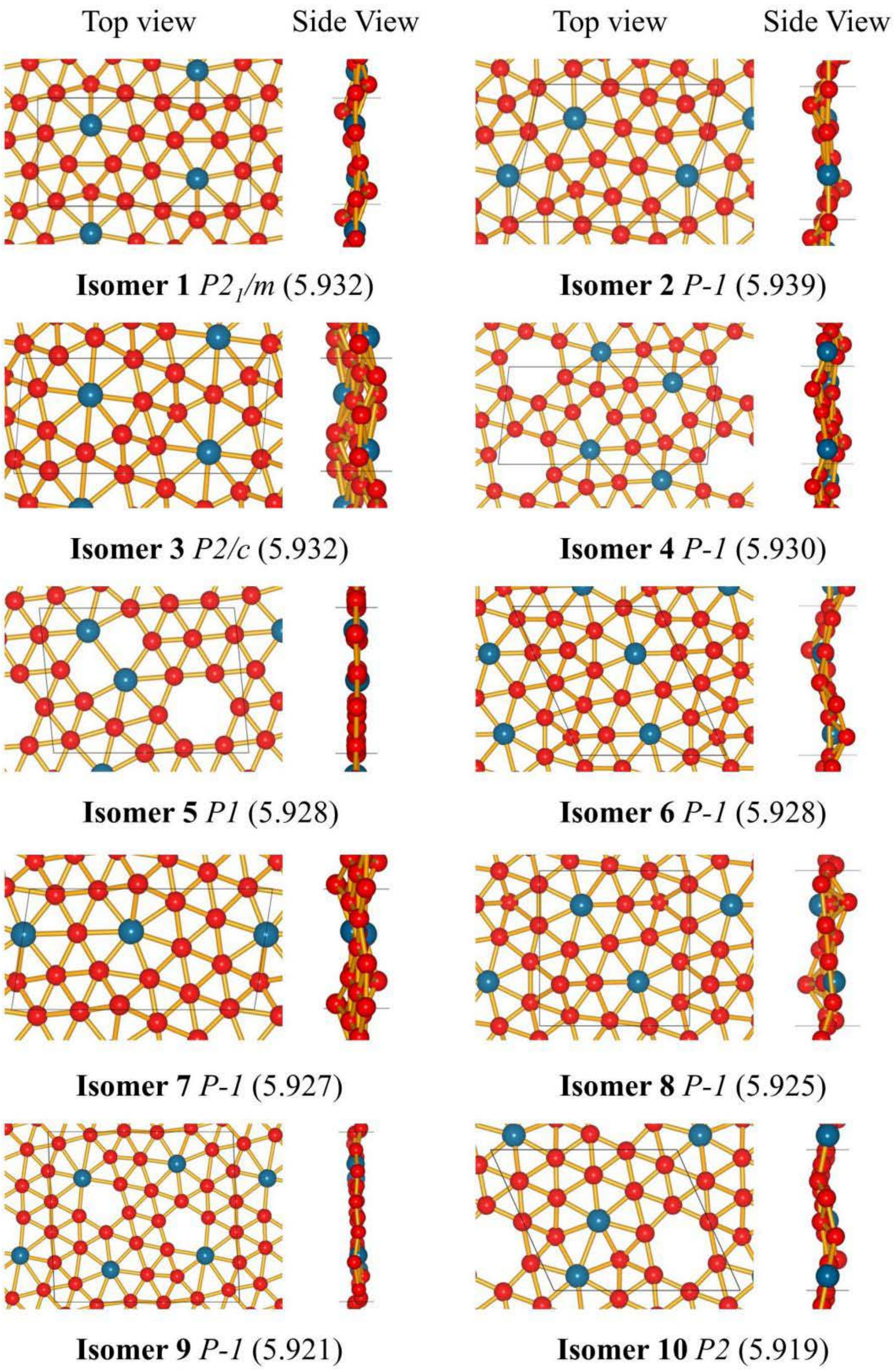

Figure S15. Optimized geometries, space groups and cohesive energies (eV/atom) of the first 10 low-lying Ni_2B_{14} sheets.

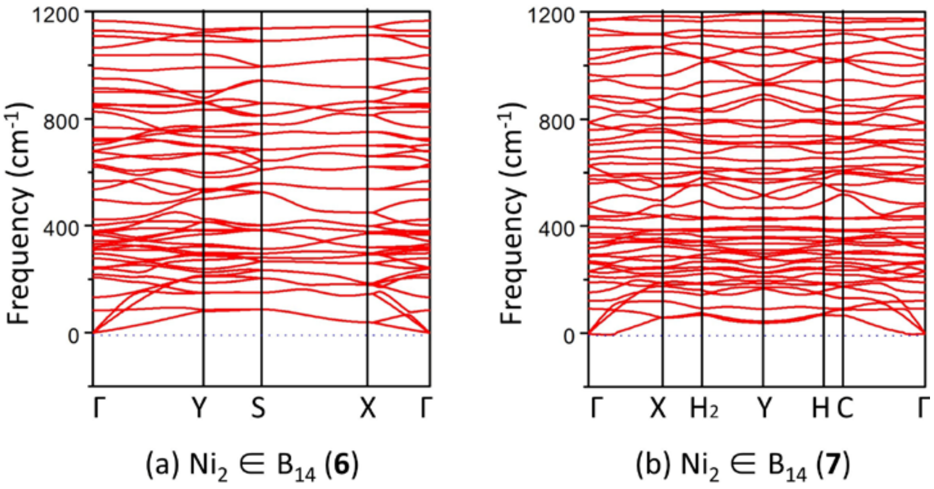

Figure S16. The phonon dispersion curves of 2D (a) heteroborophenes $Ni_2 \in B_{14}$ (**6**) and (b) $Ni_2 \in B_{14}$ (**7**).

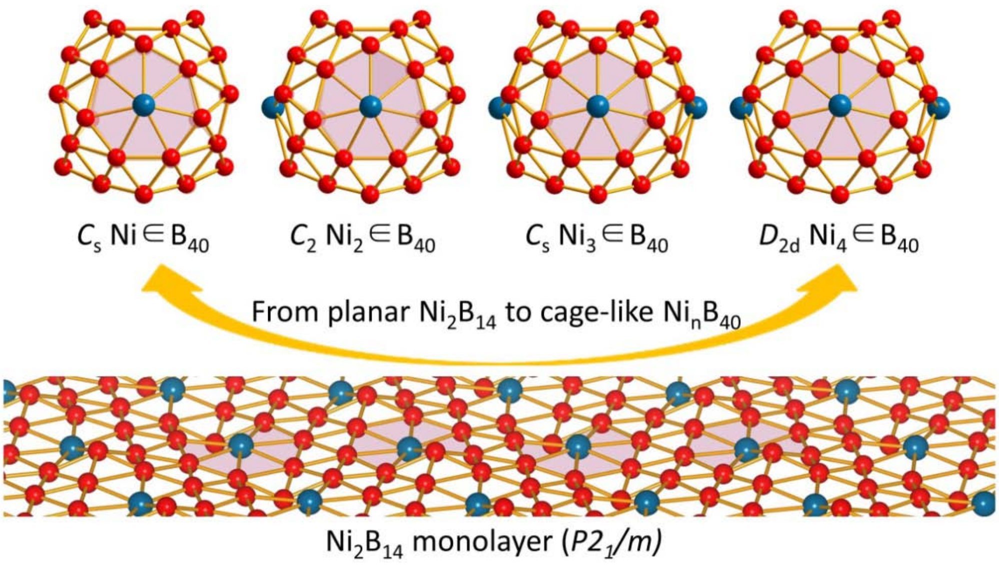

Figure S17. Cage-like heterborospherenes $Ni_n \in B_{40}$ (n=1-4) (**2-5**) and their precursor heteroborophene monolayer $Ni_2 \in B_{14}$ (**6**).

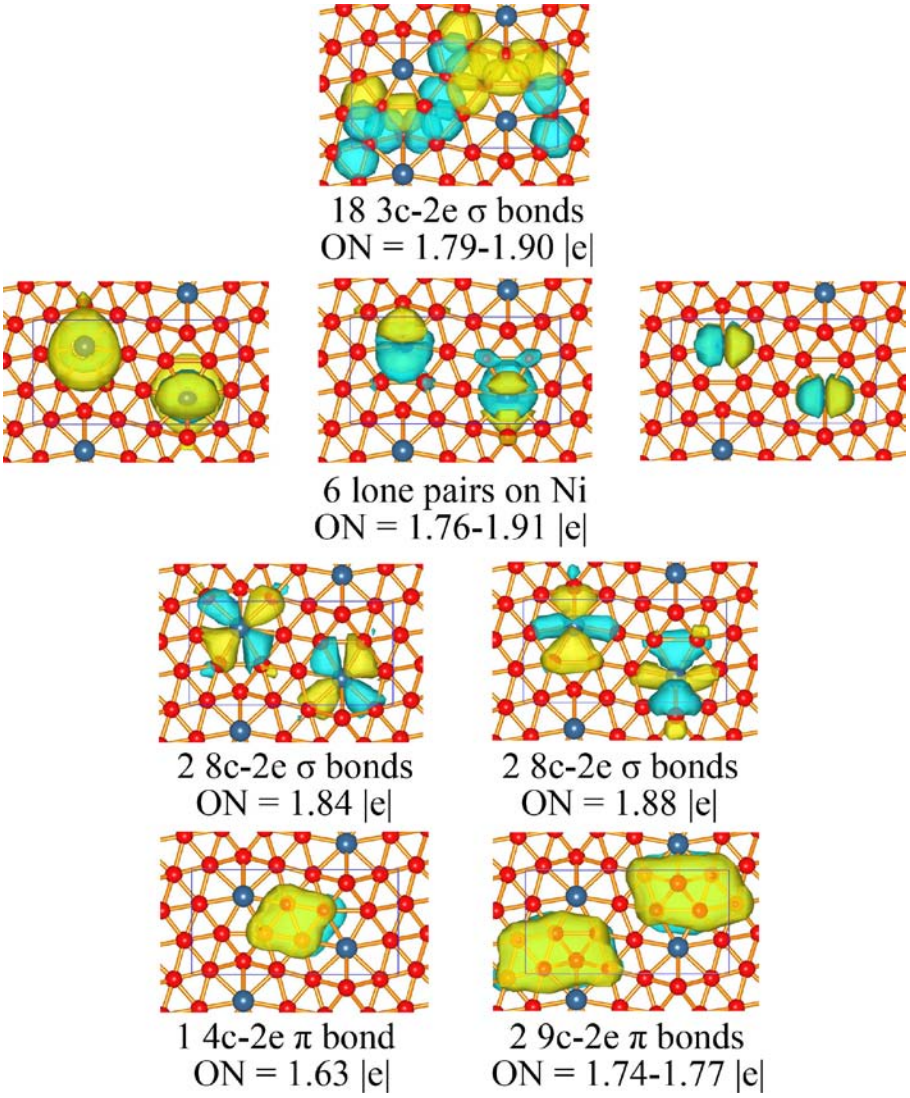
18 3c-2e σ bonds
ON = 1.79-1.90 |e|
6 lone pairs on Ni
ON = 1.76-1.91 |e|
2 8c-2e σ bonds
ON = 1.84 |e|
2 8c-2e σ bonds
ON = 1.88 |e|
1 4c-2e π bond
ON = 1.63 |e|
2 9c-2e π bonds
ON = 1.74-1.77 |e|

$Ni_2 \in B_{14}$ (**6**)

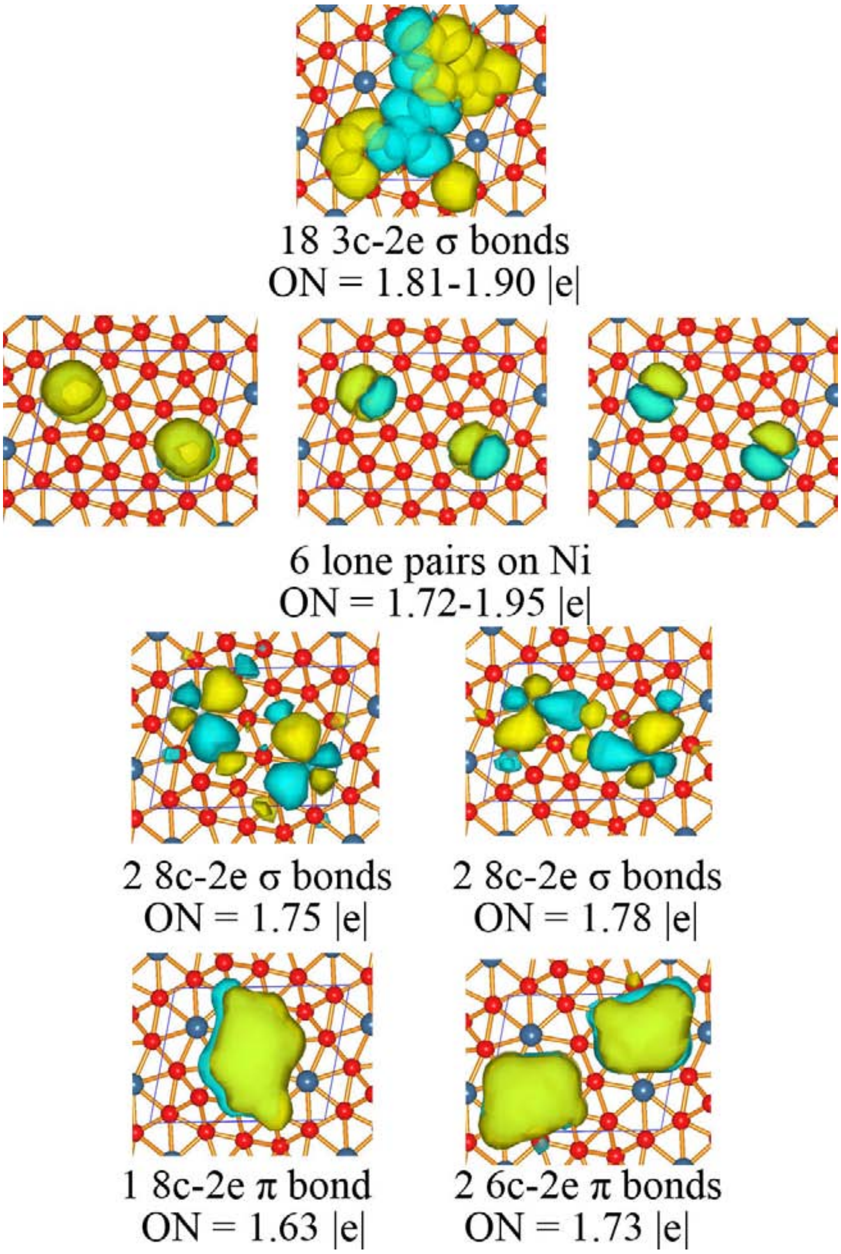

$Ni_2 \in B_{14}$ (**7**)

Figure S18. SSAdNDP bonding patterns of nanosheet $Ni_2 \in B_{14}$ (**6**) and $Ni_2 \in B_{14}$ (**7**).

Table S1. Optimized coordinates of **1**–**7**.

C_{2v} Ni∈B_{18} (**1**)

B	0.00000000	3.46096700	1.28280700
B	0.00000000	1.86244100	0.87588700
B	0.00000000	0.81249800	2.32738800
B	0.00000000	0.00000000	−1.55709400
B	0.00000000	2.88426900	−1.83181400
B	0.00000000	1.58693300	−0.83603000
B	0.00000000	3.29684100	−0.33192900
B	0.00000000	1.44826800	−2.54625800
B	0.00000000	0.00000000	−3.19522600
B	0.00000000	−1.86244100	0.87588700
B	0.00000000	−3.29684100	−0.33192900
B	0.00000000	−2.88426900	−1.83181400
B	0.00000000	−3.46096700	1.28280700
B	0.00000000	−2.40774000	2.41499500
B	0.00000000	2.40774000	2.41499500
B	0.00000000	−1.44826800	−2.54625800
B	0.00000000	−1.58693300	−0.83603000
B	0.00000000	−0.81249800	2.32738800
Ni	0.00000000	0.00000000	0.36468300

C_s Ni∈B_{40} (**2**)

B	−0.38599500	1.34552000	2.78950400
B	2.85882800	1.29172200	0.00000000
B	1.21700700	1.03777900	2.39294400
B	−0.38213000	−2.14332000	−2.72851100
B	−2.83527400	−0.37758200	−1.66544600
B	−1.99392800	2.28428400	0.00000000
B	1.67686800	−2.77256200	−0.88185100
B	−1.21869500	−2.76537800	1.40291100
B	−0.38213000	−2.14332000	2.72851100
B	2.84890800	−2.07698900	0.00000000
B	1.20319400	−1.79297200	2.37369200
B	0.39880700	2.39309200	−1.88899900
B	1.21700700	1.03777900	−2.39294400
B	1.20319400	−1.79297200	−2.37369200
B	−1.68113200	−1.28748800	−2.35693800
B	−1.65425900	0.45094100	2.39583000
B	1.67686800	−2.77256200	0.88185100
B	−1.65425900	0.45094100	−2.39583000
B	1.95606800	−0.38867100	−2.62053700
B	0.39880700	2.39309200	1.88899900
B	1.67195500	1.99913100	0.88432800
B	1.67195500	1.99913100	−0.88432800
B	−1.21869500	−2.76537800	−1.40291100
B	−1.19186900	2.02069300	1.44515800
B	−1.19186900	2.02069300	−1.44515800
B	2.52603000	−1.29790100	−1.40012600
B	2.51766900	0.47218500	−1.36647300
B	−0.38599500	1.34552000	−2.78950400
B	−2.50137000	1.02756100	−0.89361000
B	−1.98042200	−3.03167600	0.00000000
B	−1.68113200	−1.28748800	2.35693800
B	2.52603000	−1.29790100	1.40012600
B	2.51766900	0.47218500	1.36647300
B	−2.50137000	1.02756100	0.89361000
B	0.37799600	−3.13023800	−1.74574800
B	1.95606800	−0.38867100	2.62053700
B	−2.54305700	−1.78470200	−0.88439700
B	−2.83527400	−0.37758200	1.66544600
B	0.37799600	−3.13023800	1.74574800
B	−2.54305700	−1.78470200	0.88439700
Ni	−0.00660900	2.77151400	0.00000000

C_2 $Ni_2 \in B_{40}$ (**3**)

B	0.38059700	0.69539500	3.66774700
B	-2.83424900	-1.17556900	1.66643000
B	-1.21426900	0.69001700	3.17259700
B	0.41507600	-0.67885500	-2.84530100
B	2.83796200	-1.17833300	-0.69707900
B	1.95234400	-1.86352700	2.34441800
B	-1.65894000	1.09546800	-1.82781900
B	1.21095200	2.73441700	-0.18621200
B	0.39399700	3.19981200	1.24366000
B	-2.83796200	1.17833300	-0.69707900
B	-1.21426900	2.71026500	1.17067400
B	-0.39399700	-3.19981200	1.24366000
B	-1.21095200	-2.73441700	-0.18621200
B	-1.21426900	-0.68002600	-2.25852400
B	1.65894000	-1.09546800	-1.82781900
B	1.68303600	1.03376000	2.80451700
B	-1.65558900	2.31049900	-0.56107000
B	1.65558900	-2.31049900	-0.56107000
B	-2.00351400	-1.87629300	-1.39050800
B	-0.38059700	-0.69539500	3.66774700
B	-1.68303600	-1.03376000	2.80451700
B	-1.67046800	-2.29756400	1.59280000
B	1.21426900	0.68002600	-2.25852400
B	1.21426900	-0.69001700	3.17259700
B	1.21426900	-2.71026500	1.17067400
B	-2.51096500	-0.37022200	-1.10251600
B	-2.51126300	-1.65195400	0.13516200
B	0.37928900	-3.30206600	-0.14036400
B	2.51614200	-1.58532300	0.84783500
B	2.00351400	1.87629300	-1.39050800
B	1.67046800	2.29756400	1.59280000
B	-2.51614200	1.58532300	0.84783500
B	-2.53837100	0.36354700	2.12522900
B	2.53837100	-0.36354700	2.12522900
B	-0.41507600	0.67885500	-2.84530100
B	-1.95234400	1.86352700	2.34441800
B	2.51096500	0.37022200	-1.10251600
B	2.83424900	1.17556900	1.66643000
B	-0.37928900	3.30206600	-0.14036400
B	2.51126300	1.65195400	0.13516200
Ni	0.03081000	2.24346400	-1.74315600
Ni	-0.03081000	-2.24346400	-1.74315600

C_s $Ni_3 \in B_{40}$ (**4**)

B	2.51426100	-0.47081400	1.87975500
B	-0.23410700	2.84694500	1.67203400
B	2.15154100	1.16722100	1.47054500
B	-3.11916100	-0.30443900	-1.73821500
B	-2.03484300	-2.76261100	0.00000000
B	-0.36190100	-1.99394000	2.66422400
B	-1.16074100	1.69156800	-2.38173300
B	1.12060100	-1.24284300	-2.42706300
B	2.51426100	-0.47081400	-1.87975500
B	-0.23410700	2.84694500	-1.67203400
B	2.15154100	1.16722100	-1.47054500
B	-2.19110400	0.44217400	2.78600600
B	-2.68313200	1.28930700	1.43507900
B	-2.68313200	1.28930700	-1.43507900
B	-2.74668000	-1.59551600	-0.86949300
B	2.03839600	-1.70353000	0.87149200
B	0.60124700	1.65269700	-2.41761900
B	-2.74668000	-1.59551600	0.86949300
B	-2.87390500	2.02059700	0.00000000
B	1.57656900	0.37597600	2.84490700
B	0.60124700	1.65269700	2.41761900
B	-1.16074100	1.69156800	2.38173300
B	-1.80242600	-1.16403600	-2.41335000
B	1.12060100	-1.24284300	2.42706300
B	-1.80242600	-1.16403600	2.41335000
B	-1.62874100	2.56286600	-0.88268000
B	-1.62874100	2.56286600	0.88268000
B	-3.11916100	-0.30443900	1.73821500
B	-1.29504600	-2.48796500	1.43167600
B	-0.36190100	-1.99394000	-2.66422400
B	2.03839600	-1.70353000	-0.87149200
B	1.14978300	2.48021600	-0.88628900
B	1.14978300	2.48021600	0.88628900
B	0.48702400	-2.52342100	1.39386500
B	-2.19110400	0.44217400	-2.78600600
B	2.38975500	1.94413400	0.00000000
B	-1.29504600	-2.48796500	-1.43167600
B	1.27079900	-2.85785200	0.00000000
B	1.57656900	0.37597600	-2.84490700
B	0.48702400	-2.52342100	-1.39386500
Ni	-0.32049500	-0.01848400	3.17128800
Ni	-0.32049500	-0.01848400	-3.17128800
Ni	2.85803000	-0.03253300	0.00000000

D_{2d} $Ni_4 \in B_{40}$ (**5**)

B	2.84484300	1.87960500	0.40662600
B	0.00000000	1.66071200	-2.82665000
B	2.43214700	1.47199500	-1.22107000
B	-2.84484300	-1.87960500	0.40662600
B	-1.66071200	0.00000000	2.82665000
B	0.00000000	2.65389800	2.00509200
B	-0.87625800	-2.40227400	-1.65816400
B	1.47199500	-2.43214700	1.22107000
B	2.84484300	-1.87960500	0.40662600
B	0.00000000	-1.66071200	-2.82665000
B	2.43214700	-1.47199500	-1.22107000
B	-1.87960500	2.84484300	-0.40662600
B	-2.43214700	1.47199500	-1.22107000
B	-2.43214700	-1.47199500	-1.22107000
B	-2.40227400	-0.87625800	1.65816400
B	2.40227400	0.87625800	1.65816400
B	0.87625800	-2.40227400	-1.65816400
B	-2.40227400	0.87625800	1.65816400
B	-2.65389800	0.00000000	-2.00509200
B	1.87960500	2.84484300	-0.40662600
B	0.87625800	2.40227400	-1.65816400
B	-0.87625800	2.40227400	-1.65816400
B	-1.47199500	-2.43214700	1.22107000
B	1.47199500	2.43214700	1.22107000
B	-1.47199500	2.43214700	1.22107000
B	-1.40290200	-0.88799500	-2.51356400
B	-1.40290200	0.88799500	-2.51356400
B	-2.84484300	1.87960500	0.40662600
B	-0.88799500	1.40290200	2.51356400
B	0.00000000	-2.65389800	2.00509200
B	2.40227400	-0.87625800	1.65816400
B	1.40290200	-0.88799500	-2.51356400
B	1.40290200	0.88799500	-2.51356400
B	0.88799500	1.40290200	2.51356400
B	-1.87960500	-2.84484300	-0.40662600
B	2.65389800	0.00000000	-2.00509200
B	-0.88799500	-1.40290200	2.51356400
B	1.66071200	0.00000000	2.82665000
B	1.87960500	-2.84484300	-0.40662600
B	0.88799500	-1.40290200	2.51356400
Ni	0.00000000	3.17442100	0.03942300
Ni	0.00000000	-3.17442100	0.03942300
Ni	3.17442100	0.00000000	-0.03942300
Ni	-3.17442100	0.00000000	-0.03942300

Structure information of 2D heteroborophenes $Ni_2 \in B_{14}$ (**6**) and $Ni_2 \in B_{14}$ (**7**)

$Ni_2 \in B_{14}$ (**6**):

LATTICE

9.317375000000000	0.000000000000000	0.000000000000000
0.000000000000000	4.729421508568370	0.019810670355298
0.000000000000000	0.000000000000000	15.743977000000003

POSITIONS

B	0.4638767300000001	0.6735452299999991	0.4959862099999990
B	0.1450908000000000	0.3899407699999991	0.4838854399999991
B	0.5876866200000002	0.9554033999999991	0.4967939299999991
B	0.9638798300000002	0.3264569600000001	0.4963335099999990
B	0.6450853800000002	0.6100593199999991	0.5084348499999990
B	0.0876859099999999	0.0445973799999999	0.4955221200000001
B	0.5361201700000001	0.3264569600000001	0.4963335100000001
B	0.8549146199999991	0.6100593199999991	0.5084348499999990
B	0.4123140900000001	0.0445973799999999	0.4955221199999991
B	0.0361232699999999	0.6735452299999991	0.4959862099999990
B	0.3549092000000000	0.3899407699999991	0.4838854399999991
B	0.9123133799999991	0.9554033999999991	0.4967939299999991
B	0.7499999999999991	0.8736356999999991	0.5328640399999990
B	0.2500000000000001	0.1263546200000000	0.4594523399999991
Ni	0.2500000000000001	0.7476414099999991	0.4996547300000002
Ni	0.7499999999999991	0.2523621500000000	0.4926886200000001

Ni_2∈B_{14} (7):

LATTICE

5.788671637281501	-1.170228349851150	-0.019843970055151
0.000000000000000	7.532451248452662	-0.295691487343549
0.000000000000000	0.000000000000000	15.014882095600001

POSITIONS

B	0.0650039359995708	0.8965774506220131	0.2249193287214170
B	0.8585546476115479	0.4422203384906840	0.2093676905655200
B	0.6083046115954510	0.0745198822337281	0.2141165628288950
B	0.9279573708144520	0.6689012065219642	0.2214196109152880
B	0.0662377548588538	0.3047750622542801	0.2233254718494670
B	0.3858905140778560	0.8991563865425160	0.2306285199358520
B	0.1356404780617580	0.5314559302855592	0.2353773921992270
B	0.5837831636194431	0.3147668283783830	0.2043037048751680
B	0.6357539573113991	0.5516217571985591	0.2322522244465990
B	0.8098950722701860	0.2293194539965280	0.1720704477306570
B	0.4104119620538630	0.6589094403978610	0.2404413778895790
B	0.9291911896737350	0.0770988181542309	0.2198257540433310
B	0.1843000534031210	0.7443568147797230	0.2726746350340911
B	0.3584411683619150	0.4220545115776850	0.2124928583181480
Ni	0.2884228531569100	0.1411990781040950	0.2191115732037480
Ni	0.7057722725163980	0.8324771906721571	0.2256335095610000
